# Community-based non-pharmacological interventions for pregnant women with gestational diabetes mellitus: a systematic review

**DOI:** 10.1186/s12905-022-02038-9

**Published:** 2022-11-29

**Authors:** Chinonso Nwamaka Igwesi-Chidobe, Peace Chioma Okechi, Grace Nneoma Emmanuel, Benjamin C. Ozumba

**Affiliations:** 1 Department of Medical Rehabilitation, Faculty of Health Science and Technology, College of Medicine, University of Nigeria Enugu Campus, Nsukka, Nigeria; 2 Global Population Health (GPH) Research Group, University of Nigeria, Nsukka, Nigeria; 3 Department of Obstetrics and Gynaecology, Faculty of Medicine, College of Medicine, University of Nigeria, Nsukka, Nigeria

**Keywords:** Community-based, Non-pharmacological, Gestational diabetes mellitus, Systematic review, Meta-analysis, Pregnant women

## Abstract

**Background:**

Non-pharmacological interventions are the first line of Gestational diabetes mellitus (GDM) management. Community-based interventions are cheaper, more accessible, with higher patient satisfaction.

**Objectives:**

To systematically review community-based non-pharmacological interventions and evaluate their effectiveness for GDM.

**Search strategy:**

Twelve bibliographic databases and reference list of related studies from inception until January 2022.

**Selection criteria:**

All primary studies of community-based non-pharmacological interventions for GDM reported in English which investigated any behavioural or clinical outcome(s).

**Data collection and analysis:**

Data were extracted using modified Cochrane’s data extraction template. Studies were evaluated using Cochrane Collaboration’s risk of bias tool. Narrative synthesis was used to summarise findings. This study is registered with PROSPERO (CRD42021257634).

**Main results:**

Twenty-seven studies involving 6,242 pregnant women with GDM investigated self-management programmes, medical nutrition/diet therapy, exercise/physical activity, combined diet and exercise, calcium plus vitamin D supplementation, and continuous glucose monitoring. Self-management programmes were more effective than routine care in improving self-efficacy, two-hour postprandial blood glucose, and lifestyle behaviours but were as effective as routine care in improving infant birth weight. Self-management programmes were superior to or as effective as usual care in improving fasting blood glucose, blood glucose control, glycated haemoglobin, macrosomia, and preterm delivery. Medical nutrition/diet therapy was more effective than usual care in improving postprandial blood glucose levels. Postprandial blood glucose levels were better improved by regular supervised exercise plus daily brisk walks or a daily walking intervention than routine obstetric care or no treatment. The effects of exercise/physical activity programmes were mostly inconsistent for other outcomes. Diet and exercise were superior to diet alone in reducing maternal weight gain although there were similar outcomes for other pregnancy and foetal outcomes. Limited or conflicting evidence was found for other outcomes and interventions including calcium and vitamin D supplementation and continuous glucose monitoring intervention.

**Conclusions:**

Community-based non-pharmacological interventions are more effective than placebo; and are more or as effective as usual care. Self-management programmes and medical nutrition/diet therapy had the most promising GDM outcomes.

**Funding:**

There was no funding for this study. The study design, data collection, data analysis and interpretation, and writing of this manuscript were not influenced externally by any funder.

**Supplementary Information:**

The online version contains supplementary material available at 10.1186/s12905-022-02038-9.

## Introduction

Gestational diabetes mellitus (GDM) is diagnosed by the detection of hyperglycaemia during pregnancy. This can convert a natural process to one fraught with increased risk for foetal and maternal mortality and morbidity, an increased need for surveillance, increased emotional distress, and reduced quality of life [1, 2]. Five percent of women develop type 2 diabetes within 10 years following first onset of GDM [3–5]. The prevalence of GDM is 1% – 30% globally. The burden is greatest in the Middle East and some North African countries (8.8–20.0%), followed by South- East Asia (9.6–18.3%), the Western Pacific (4.5–20.3%), South and Central America (7.1–16.6%), sub-Saharan Africa (8.5–13.1%) and North America and the Caribbean (6.5–11.9%). The lowest burden of GDM is found in Europe, and this region also has the widest variation in prevalence (1.8–31.0%) [2, 6].

GDM results from carbohydrate intolerance or hyperglycemia with first onset occurring in a present pregnancy. Most GDM are detected in late second trimester (13–26 completed weeks of gestation) or early in the third trimester (27–40 weeks). It appears that women who developed GDM in late gestation had decreased peripheral insulin sensitivity (insulin sensitivity – ability of insulin to increase glucose uptake in skeletal muscle and adipose tissues) even before conception. There is defect in the pancreatic β- cell function such that as pregnancy progresses, insulin resistance increases, the insulin response subsequently becomes inadequate resulting in hyperglycaemia. Inflammation, which has been linked to obesity, is also important in the pathophysiology of GDM as it disrupts insulin signaling. Pancreatic β- cell dysfunction can progress after the first GDM pregnancy and appear to be linked with retention of excessive gestational weight gain and increases in insulin resistance. Decreased insulin sensitivity affect maternal amino acid and lipid metabolism, which increases foetal growth and adiposity, and this can be associated with complications during delivery. Other complications in the offspring include congenital malformations, shoulder dystocia, respiratory distress syndrome, neonatal hypoglycemia, hyperbilirubinaemia, and hypocalcaemia [2, 7, 8].

Risk factors for GDM include body mass index (BMI) greater than 25kg/m^2^, advanced maternal age, non-white ancestry, family history of type 2 diabetes mellitus, previous history of GDM, parity, male foetus, multiple pregnancy, genetic factors, polycystic ovarian syndrome, cigarette smoking, psychosocial factors such as depression during pregnancy, unhealthy dietary behaviour (‘western diet style – high sugar consumption including sweets, sweetened beverages, potatoes; increased dietary fat consumption including fried foods and animal fat; and protein including red/processed meat; refined grain products e.g., French fries and pizza) before and during pregnancy, and physical inactivity before and during pregnancy. Furthermore, environmental (e.g., persistent organic pollutants and endocrine disruptors), and psychosocial (e.g., depression in the first and second trimester) factors increase the risk of developing GDM. Together with genetic susceptibility, these factors may explain the global differences in GDM burden. Nearly half of all GDM cases can be prevented by adopting a healthy diet prior to pregnancy (large uptake of fibre, nuts, fruits, green leafy vegetables, poultry and fish, an overall ‘Mediterranean’ diet), maintaining a BMI <25 kg/m^2^, doing exercise ≥30 minutes per day, and avoiding smoking [2, 3, 6]. Improvement in the metabolic processes associated with GDM occurs in women who return to pre-pregnancy body weight; which can be facilitated by engagement in physical activity and adoption of healthy diet [2].

Treatment of GDM aims to prevent foetal overgrowth and pregnancy complications and involves non-pharmacological and pharmacological modalities. Pharmacological treatments are utilized when non-pharmacological interventions do not achieve glycaemic control. Insulin therapy is the main pharmacological intervention, but it is associated with discomfort, increased cost, and risk of hypoglycaemia, requiring regular hospital visits and dose adjustments. Hence, glucose lowering oral medications such as metformin are becoming popular. Non-pharmacological interventions include mainly diet and physical activity [2] and are often sufficient to achieve glycaemic control [9, 10]. Indeed, lifestyle interventions which promote healthy diet and physical activity are also effective in the prevention of GDM. Furthermore, 70-90% of pregnant women with GDM can be effectively managed with lifestyle interventions alone [11]. Psychological and health education interventions may be relevant where they promote health behaviour changes such as self-monitoring of blood glucose levels [12–14]. A randomised controlled trial (RCT) showed improvements in 1-hour postprandial blood glucose after an educational intervention. The study reported no statistically significant differences amongst four groups of nutrition therapy only, nutrition therapy plus education, insulin therapy only, and insulin therapy plus education in other outcomes such as fasting blood glucose, 2-hour postprandial blood glucose, glycated haemoglobin and quality of life [15]. However, this study may have been underpowered to detect statistical significance. Self-management programmes usually contain psychological and health education components, and systematic reviews have suggested that they can be effective in the management of GDM [16–18].

Community-based treatment is associated with lower costs, higher patient satisfaction, fewer hospitalizations and emergency department visits, and lower mortality [19, 20]. Community-based interventions are often outside large health institutions such as primary health care and general community settings, can involve non-medical personnel, have behavioural expectations and active participation, are culturally sensitive rather than routine, and are usually not intrusive [21]. Community-based interventions can be seen as multicomponent interventions that combine individual and environmental change strategies across multiple settings aiming to prevent diseases and to promote well-being among population groups outside of mainstream secondary and tertiary health facilities [22].

There is no synthesized evidence on community-based non-pharmacological interventions for GDM. Therefore, we aimed to systematically summarise the evidence regarding community-based non-pharmacological interventions for GDM, including intervention content and effectiveness. We reported this systematic review in line with the Preferred Reporting Items for Systematic reviews and Meta-Analyses (PRISMA) 2020 and the Synthesis without meta-analysis (SWiM) in systematic reviews reporting guidelines [23, 24].

## Methods

Eligibility criteria, information sources, selection process, data items, assessment of reporting bias and certainty assessment are detailed in Table 1.

**Table 1:** Processes utilised in selecting and appraising studies

**Eligibility criteria**	**Information sources**	**Selection process**	**Data items**	**Assessment of reporting bias**	**Certainty assessment**
We included all primary studies RCTs, non-RCTs, pre-test/post-test studies, observational studies, and qualitative studies) published in English, that investigated all types of community-based non-pharmacological interventions for pregnant women with GDM. We defined ‘community’ as settings outside of tertiary and secondary health facilities including primary health care centers, outreach centers, schools, churches, small community clinics within rural or urban areas. We defined non-pharmacological interventions as that do not involve the use of medications and surgery as treatment. We included interventions administered by anyone (including health professionals, alternative practitioners, or peers). All intervention delivery modes (face-to-face, telephone-based, web-based, etc.) were eligible for inclusion. There was no restriction of studies in relation to control groups and timing of assessment or intervention delivery. The primary outcome of this review is change in any health behaviour. The secondary outcomes include all clinical outcomes such as blood glucose levels, insulin sensitivity, gestational hypertension, preeclampsia, etc. We excluded publications without primary data or duplicate publications.	The Health Inter-Network Access to Research Initiative (HINARI) platform was used to search PubMed, CINAHL, CENTRAL, Global Index Medicus, African Index Medicus, African Journal Online, WPRIM (Western Pacific Region Index Medicus), LILACS (Latin American and Caribean Centre on Health Science Information), IMSEAR (Index Medicus for South-East Asia Region), IRIS (WHO digital publications), BLDS (British Library for Development Studies) from inception until August 2020. Additional studies were located from Google scholar and the reference list of relevant studies and systematic reviews. An update to all searches was done covering the period from September 2020 to January 2022.	Literature search results were imported into Mendeley and deduplicated before exportation into Microsoft Excel 2007 to facilitate the management of articles and selection of studies for inclusion into the review based on the eligibility criteria. Screening was done in two stages. Firstly, two trained reviewers (PCO, GNE) independently screened the title and abstracts of the retrieved studies and identified studies that apparently met the eligibility criteria. Disagreements at this stage were resolved by discussion between the two reviewers and forwarding the full texts of such studies to the second stage of screening. Secondly, PCO and GNE independently read through the full texts of selected studies and identified those that were eligible for the review. Disputes at this stage was resolved by the third reviewer (CNI-C). Details of study selection are presented in a flow chart (Fig. 1).	Study citation (authors’ names and year), the country where the study was conducted, participants’ characteristics (age, occupation and education), sample size, study design, intervention description, who delivered the intervention, intervention duration, intervention follow-up, attrition, results of outcome(s) assessed, and the risk of bias in each study were collected from included studies.	We checked if the rating of bias, study design and the size of study influenced consistent and inconsistent outcomes reported by more than one study.	We used the principles of Grading of Recommendation, Assessment, Development and Evaluations (GRADE) to assess the overall strength of evidence for each outcome based on inconsistency, imprecision, indirectness, and publication bias. Overall strength of evidence was rated as very low (very uncertain effect estimates), low (effect estimates will very likely change with further research), moderate (effect estimates will likely be affected by further research), and high (effect estimates unlikely to change with further research).

### Search strategy

Search strategies for the twelve databases were informed by the Cochrane handbook for systematic reviews of interventions [25]. In line with the PRISMA 2020 guidelines [23, 26], searches involved several combinations of MeSH and free text terms and word variants for pregnant, gestational diabetes, community-based and non-pharmacological interventions. Search strategies were developed for the different databases and was piloted to establish sensitivity prior to searching (appendix 1).

### Data collection process

Data were collected using a data extraction form that was adapted from the Cochrane Group’s Data Extraction Template [27]. Three reviewers (CNI-C, PCO, GNE) independently piloted the form on a random sample of 5 articles. Final amendments were made to the form prior to data extraction. PCO and GNE independently extracted data from studies. Inconsistencies in extracted data were resolved by discussion with CNI-C. A maximum of three email requests were made to corresponding authors of studies with missing or unclear information.

### Study risk of bias assessment

Two reviewers (CNI-C, PCO) independently assessed the risk of bias in all RCTs. Other study designs were regarded as high risk of bias. The risk of bias in RCTs were assessed using the Cochrane’s risk of bias tool including selection bias, performance bias, detection bias, attrition bias, reporting bias and other potential threats to validity [28]. For each RCT, low risk of bias in all domains was classified as low risk of bias overall. High risk of bias or unclear risk of bias in only one item was classified as minimal risk of bias overall. High risk of bias or unclear risk of bias in two to three items was classified as moderate risk of bias overall. High risk of bias or unclear risk of bias in four items and above was classified as high risk of bias overall.

### Synthesis methods

Narrative synthesis was used due to clinical, methodological, and statistical heterogeneity arising from diverse interventions, study designs, methods, and outcomes, respectively.

This systematic review is registered with PROSPERO (CRD42021257634) and the protocol is available online.

## Results

### Study selection

**Fig. 1:**
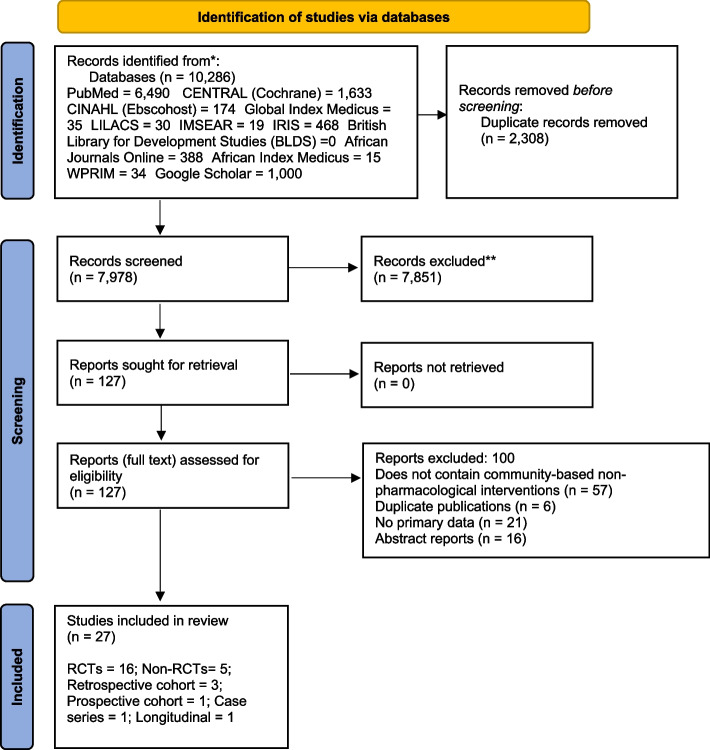
Flow diagram of the study selection process

One thousand eight hundred sixteen duplicates were removed from the initial search yield of 9 162. 7 346 titles and abstracts were screened, and 109 full text articles were screened. 25 articles were eligible. Two RCTs were included from the update search. A total of 27 articles were included in this review: 16 RCTs and 11 quasi-experimental studies (Fig. 1).

### Study characteristics

Included study characteristics are illustrated in Table 2. Identified studies were published between 2004 and 2021; and involved 6,242 pregnant women with gestational diabetes mellitus in China (6), Iran (5), United State of America (3), Canada (2), Australia (1), Brazil (1), Croatia (1), Egypt (1), Ireland (1), Japan (1), Mexico (1), Nigeria (1), Oman (1), Thailand (1), and Turkey (1). The community-based non-pharmacological interventions were delivered in primary health care centres (antenatal/maternal clinics) (8), private community clinics (5), obstetric community clinics (3), gestational diabetes mellitus community clinics (3), perinatology community clinics (2), outpatient community clinics (2), medical centre (1), and urban community clinic (1) by researchers (9), dieticians/nutritionists (4), physicians and researchers (2), physicians (2), nurses (2), physiotherapists (1), educators (1), physician and nurses (1), educator and physicians (1), exercise physiologist and nurse (1), midwives and nurses (1), dietician and nurse (1), not specified (1). The control interventions were delivered in the same facilities.

**Table 2:** Characteristics of studies of pregnant women with gestational diabetes

**Author (year)**	**Country**	**Participant characteristics**			**Study sample size**	**Study design**	**Who delivered the intervention**	**Intervention duration and follow-up periods**	**Attrition rate**
		Age (range or average in years)	Occupation	Education					
**Daniel et al.,** [29]	Nigeria	I=32 C=32·9	NS	NS	34; I=17 C=17	RCT	Physiotherapists	8 weeks	4 (I=2; C=2)
**de Barros et al.,** [30]	Brazil	18-45	NS	NS	64; I=32 C=32	RCT	Researcher	Recruitment (gestational age ranging from 24 to 34 weeks) to end of gestation	0
**Kokic et al.,** [31]	Croatia	20-40	NS	Secondary: (I=7, C=7); Tertiary: (I=11, C=13)	42; I=20, C=22	RCT	Physicians	6 weeks (Minimum)	4
**Mohebbi et al.,** [32]	Iran	17-41	NS	I: (illiterate =5, High school=21, diploma=20, university=9); C:(illiterate=9, High school=21, diploma=12, university=13)	110: I=55 C=55	RCT	Educator	6 months	0
**Yang et al.,** [33]	China	18-45	NS	NS	157: I=(A=57; B=50) C=50	Non-randomized controlled design	Obstetrician, Researchers	NS	0
**Ibrahim & Saber,** [34]	Egypt	≥ 18 - ≤ 40	I: (not working=24, working=16); C: (not working=32, working=8)	I: (not educated =2, intermediate education=20, university / academic=18); C: (not educated=14, intermediate education=18, university/ academic=8)	80; I=40, C=40	Non-randomized controlled design	Researcher	NS	0
**Bartholomew et al.,** [35]	USA	≥18	NS	51·4% College Graduates; 24·3% completed some college; 20·3% completed 12th grade, graduated high school, or earned a general education diploma; and 4·1% had < a 12th-grade education.	100; I=50, C=50	RCT	Maternal Foetal Medicine Physician and Nurses	6 weeks	26
**Yu et al.,** [36]	China	28-36	NS	NS	340; I=150, C=190	Prospective Cohort Study	Research Nurses	5 weeks	3
**Sunsaneevithayakul et al.,** [37]	Thailand	Over 30	NS	NS	54	Case series	Diabetes nurse educator and physicians	3 days	0
**Hu et al.,** [38]	China	I:30·3 ± 4·9; C:29·7 ±3·7	NS	NS	140; I=66 C=74	RCT	Dietitians	5 days	0
**Louie et al.,** [39]	Australia	26-42	NS	NS	99: LGI=50; HF=49	RCT	Endocrinologist	NS	7
**Asemi et al.,** [40]	Iran	18-40	NS	NS	58; I=29, C=29	RCT	NS	4 weeks; Follow-up: once a week	6; I=3, C=3
**Grant et al.,** [41]	Canada	18-45	NS	NS	47; I=24, C=23	RCT	Dietitian	8 weeks	4
**Artal et al.,** [42]	USA	Over 18	NS	NS	96; ED=39, D=57	Non-randomised controlled trial	Exercise physiologist, Diabetes nurse Dietitian	NS	8; ED=2, D=6
**Kgosidialwa, et al.,** [43]	Ireland	I= 33·4±4·9 C=31·5±5·2	NS	NS	3066; I=567, C=2499	Retrospective Cohort	Midwife and diabetes nurse	NS	0
**Wang et al.,** [44]	China	22-35	NS	High school education (81·7 %)	14168; GDM-E=2061; GDM-nE=689; non-GDM =11418	Retrospective cohort	Researchers	6 months	0
**Mendelson et al.,** [45]	USA	18-40	NS	NS	100; I=49, C=51	RCT	Nurses	NS	0
**Ural & Beji,** [46]	Turkey	18 or older	I= (Housewife=30, 65·2%; Civil servant, worker =16, 34·8%; Employed = 7, 15·2%; Unemployed = 39, 84·8%); C= (Housewife = 34, 81%; Civil servant, worker = 8, 19%; Employed = 6, 14·3%; Unemployed = 36, 85·7%)	I= (Primary= 20, 43·5%; Secondary = 19, 41·3%; Higher education=7, 15·2%); C= (Primary school=28, 66·7%; Secondary school=9, 21·4%; Higher education=5, 11·9%)	100; I=50, C=50	RCT	Researchers	Intervention duration: 3 days. Follow-up: 2 weeks after the education program and 2nd week of the postpartum.	12; I=4, C=8
**Kolivand et al.,** [47]	Iran	I=32·4 ± 5·6 C= 30·2 ± 4·5	I= (Housewife = 57, 76%; Employed = 18, 24%; C= (Housewife = 65, 85·5%; Employed = 11, 14·5%)	I= (Elementary =1, 1·3%; Secondary=24, 32·0%; Diploma=26, 34·7%; University= 24, 32·0%); C= (Elementary =20, 26·3%; Secondary=11, 14·5%; Diploma=32, 42·1%; University= 13, 17·1%)	152; I=76, C=76	RCT	Researcher	Intervention duration: 7 weeks, Follow-up: 1 month	1
**Al Hashmi,** [48]	Oman	19-43	Medical Professional = 16, 32·6%; Teaching Professional = 11, 22·4%; Administrative Professional = 17, 34·7%; Business / Engineering / IT Professional = 9, 15·3%. (Working: 49, 54·4%; Not working: 41, 45·6%)	Less than high school=13, 14·4%; High school graduate=30, 33·3%; Some college/College graduate=35, 44·4%; Graduate degree=7, 7·8%	90; I=45, C=45	RCT	Researcher	4 weeks	0
**Shi et al.,** [49]	China	21-44	I: (Mental = 169, 55·05%; Manual = 53, 17·26%; Home = 85, 27·69): C: (Mental = 103, 56·91%; Manual =29, 16·02%; Home = 49, 27·07%)	I: (High=233, 75·90%; Middle=45, 14·66%; Low=29, 9·45%); C: (High=149, 82·32%; Middle=18, 9·94%; Low=14, 7·73%)	488; I=307, C=181	Retrospective Cohort	Nutritionists	First follow-up: within 2 weeks since the initial diagnosis of GDM and the start of MNT. Further follow-up visits every 2-4 weeks	0
**Perichart-Pereraet al.,** [50]	Mexico	19-43	NS	NS	78; I=39, C=39	Non-randomised controlled trial	Dietitian	Follow up: with every 2 weeks until delivery	0
**Murphy et al.,** [51]	Canada	NS	NS	Individual method: Elementary = 0, High school = 9 (26%), Post-secondary = 26 (74%); Small group method: Elementary school = 0, High school = 6 (15%), Post-secondary = 33 (85%)	76: Individually= 35, Small groups= 41	Non-randomised controlled trial	Dietitian and Nurse	Intervention duration: 1 hour, follow-up: 1 week	0
**Hayashi et al.,** [4]	Japan	29-42	NS	NS	73	Longitudinal	Researchers	7-12 weeks	49
**Karamali et al.,** [52]	Iran	18-40	NS	NS	60; I=30, C=30	RCT	Researchers	6 weeks	0
**Tian et al.,** [53]	China	18-45	NS	NS	309; I= 147 C= 162	RCT	Physicians and Researchers	Every 2 weeks until delivery	23
**Barati et al.,** [54]	Iran	18-35	Employed (88·7%) Housewife (11·3%)	University (37·7%) Diploma (34·0%) No diploma (28·3%)	112; I= 56 C= 56	RCT	Researcher	4 weeks	7

*NS* Not specified, *I* Intervention group, *C* Control group, *USA* United States of America, *RCT* Randomized Controlled Trial, *HBM* Health Belief Model, *SMBG* Self-monitoring of Blood Glucose, *LGI* Low Glycemic Index, *HF* High Fibre, *ED* Exercise & Diet, *D* Diet, *GWG* Gestational Weight Gain, *DSCA* Diabetes Self-Care Activities Measure, *DMSES* Diabetes Management Self-Efficacy Scales, *HPLP II* Health-Promoting Lifestyle Profile II, *SF-36* Short Form 36 Health Survey, *CES-D* Center for Epidemiologic Studies Depression Scale, *BMI* Body mass index, *FBG* Fasting blood glucose, *CGMS* Continuous glucose monitoring system, *I* Intervention, *C* Control, *GDM-E* Gestational Diabetes Mellitus-Exercise, *GDM-nE* Gestational Diabetes Mellitus-non Exercise, *non-GDM* Non-Gestational Diabetes Mellitus, *HbA1c* Glycated haemoglobin

### Risk of bias in studies

Table 3 highlights the risk of bias in individual RCTs. Overall, two RCTs were low risk of bias, two RCTs were minimal risk of bias, eight RCTs were moderate risk of bias, and three RCTs were high risk of bias. All eleven quasi-experimental studies were regarded as high risk of bias.

**Table 3:** Risk of bias table for the randomised controlled trials (RCTs)

**Author’s name and year**	**Random sequence generation (selection bias)**	**Allocation concealment (selection bias)**	**Blinding of participants and personnel (performance bias)**	**Blinding of outcome assessment (detection bias)**	**Incomplete outcome data (attrition bias)**	**Selective reporting (reporting bias)**	**Other biases**	**Overall grading of bias**
Karamali et al. [52]	Low risk	Low risk	Low risk	Low risk	Low risk	Low risk	Low risk	Low risk
Louie et al., [39]	Low risk	Low risk	Low risk	Low risk	Low risk	Low risk	Low risk	Low risk
de Barros et al., [30]	Low risk	Low risk	Low risk	Low risk	Low risk	Low risk	Low risk	Low risk
Kolivand et al., [47]	Low risk	Low risk	Low risk	Unclear	Low risk	Low risk	Low risk	Minimal risk
Kokic et al., [31]	Low risk	Low risk	High risk	Low risk	Low risk	Low risk	Low risk	Minimal risk
Tian et al., [53]	Low risk	Unclear	Low risk	Unclear	Low risk	Low risk	Low risk	Moderate risk
Barati et al., [54]	Low risk	Low risk	High risk	Unclear	Low risk	Low risk	Low risk	Moderate risk
Mohebbi et al., [32]	High risk	Low risk	Unclear	Low risk	Low risk	Low risk	Low risk	Moderate risk
Al Hashmi, [48]	Low risk	Unclear	High risk	Low risk	Low risk	Low risk	Low risk	Moderate risk
Bartholomew et al., [35]	Low risk	Low risk	High risk	Low risk	High risk	Low risk	Low risk	Moderate risk
Asemi et al., [40]	Low risk	Unclear	Unclear	Unclear	Low risk	Low risk	Low risk	Moderate risk
Grant et al., [41]	Low risk	Low risk	Unclear	Unclear	Low risk	Low risk	High risk	Moderate risk
Mendelson et al., [45]	Low risk	High risk	High risk	High risk	Low risk	Low risk	Low risk	Moderate risk
Daniel et al., [29]	Unclear	Unclear	Unclear	Unclear	Low risk	Low risk	Low risk	High risk
Hu et al., [38]	Unclear	Unclear	Unclear	Unclear	Low risk	Low risk	Low risk	High risk
Ural & Beji, [46]	High risk	High risk	High risk	High risk	Low risk	Low risk	Low risk	High risk

### Results of individual studies

Table 4 presents the description of the community-based non-pharmacological interventions and the control group interventions as well as the results of each study.

**Table 4:** Results of each individual study included

**S/N**	**Author’s name and year**	**Overall risk of bias**	**Community-based non-pharmacological interventions and duration**	**Control group interventions and duration**	**Results in favour of experimental (community-based non-pharmacological) interventions**	**Results in favour of control group interventions**	**No difference in the outcomes of both groups –**
**1**	Karamali et al. [52]	Low risk	**Calcium and vitamin D** supplements. 1000 mg Ca/d and two pearls containing 1250 μg (50 000 IU) of cholecalciferol (vitamin D3) during the intervention (one at study baseline and another at day 21 of the intervention)	Two **placebos** of vitamin D at the mentioned times and placebos of Ca every day for 6 weeks.	Significant decrease in **caesarean section rate** (23·3% vs 63·3%, *P* = 0·002) and **maternal hospitalization** (0 vs 13·3%, *P* = 0·03) compared with those receiving placebo **Newborns of GDM** women randomized to Ca + vitamin D had no case of **macrosomia**, while the prevalence of macrosomia among those randomized to placebo was 13·3% (*P* = 0·03). Lower rates of **hyperbilirubinaemia** (20·0% v. 56·7%, *P* = 0·03) and **hospitalization** (20·0% vs 56·7%, *P*= 0·03) were also seen in the supplemented group of newborns than in the placebo group.	–	–
**2**	Louie et al., 2011 [39]	Low risk	**Low–glycaemic index (LGI) diet**: low GI (target GI ≤ 50) healthy diets of similar protein (15–25%), fat (25–30%), and carbohydrate (40–45%) content	**Conventional high-fibre diet**: moderate GI (target GI ≈ 60) healthy diets of similar protein (15–25%), fat (25–30%), and carbohydrate (40–45%) content	–	–	LGI diet and a conventional HF diet produced similar pregnancy outcomes. Average infant birth weight, birth weight centile, and ponderal index were within healthy norms in both groups. Fewer women in the LGI group gained an excessive amount of weight but this was not statistically significant (LGI 25% vs. HF 42%; *P* = 0·095). Compliers in the LGI group appeared to gain less weight than those in the HF group although this was not statistically significant (LGI 11·2±0·9 kg vs HF 13·7±1·0 kg; *P* = 0·073). No significant difference in foetal abdominal circumference at 36-37 weeks’ gestation (mean ± SEM LGI 327·6 ± 19·2 mm vs HF 322·6 ± 14·6 mm; *P* = 0·186).
**3**	de Barros et al., 2010 [30]	Moderate risk	**Exercise** group underwent a **resistance exercise** programme until the end of gestation PLUS routine prenatal care PLUS systematic evaluation and diabetic dietary instructions from a nutritionist. Resistance exercise was performed with an elastic band. The patients were advised to start the program about 90 minutes after consuming a meal of their preference (breakfast, lunch, or dinner) and after the measurement of capillary glycemia with an Accu-Chek Advantage glucose meter (Roche Diagnostics, Indianapolis, IN). If capillary glucose levels were between 100 and 250 mg/dL, exercise group patients started the program with a stretching sequence. If capillary glycemia was below this range, the patients were instructed to do the resistance exercise program on the next day to prevent hypoglycemia. If the values were above this range, the patients were instructed not to undergo resistance exercise to prevent the occurrence of ketoacidosis and to contact the responsible obstetrician. The resistance exercise program consisted of a circuit type resistance training, elaborated in such a way that the main muscle groups of the patients would be exercised (chest, back, biceps, triceps, deltoid, quadriceps, thigh, and calf muscles). A circuit series was defined as a sequence of these eight exercises (stations). The women performed 15 repetitions of each exercise (station), with a minimum resting period of 30 seconds and a maximum of 1 minute between each one. In the first and second week of follow-up, the women underwent 2 circuit series, followed by 3 circuit series from the third week of inclusion in the study to the end of gestation. Exercise intensity was controlled by the women themselves using a perceived exertion scale for resistance exercise provided to them. The women were advised to maintain an exercise intensity close to 5 or 6, which corresponds to a “somewhat heavy” exercise perception. The patients received written guidelines of how to perform each exercise and were instructed to undergo the program on 3 non-consecutive days a week (twice a week at home). The participants were contacted by telephone at least once a week to verify adherence to the program. The other session was performed during the weekly return visit, always under the supervision of the lead researcher. On that occasion, the patients were asked whether they had performed any type of physical activity other than resistance exercise. In addition, the adequate execution of resistance exercise at the correct intensity was verified.	**Routine** prenatal care PLUS systematic evaluation and diabetic dietary instructions from a nutritionist.	A significant decrease in the number of patients who required insulin was observed in exercise group compared with control group. Glycemic control was significantly better in exercise group compared with control group. In exercise group, the percentage of weeks spent within the target glucose range (80% of weekly capillary glucose measurements within preestablished guideline values) was significantly higher when compared with control group. Newborn birthweight greater than 4000 g was observed in 1 EG case and 3 CG cases.	–	No significant difference in mean (SD) glucose levels was observed between exercise and control groups. Mean glucose levels measured by the patients at different times of the day throughout the follow-up period were lower in exercise group compared with control group, but the difference was not significant. The 2 groups did not differ significantly in terms of the amount of insulin (international units per kilogram) required by the patients or in the time interval (weeks) between inclusion in the study and insulin use. Exercise group patients who used insulin continued to present adequate glycemic control according to the target established for a longer percent period of weeks than control patients who used insulin (EG: 0·40 (0·24) vs CG: 0·25 (0·23), but the difference was not significant (*P*=0·173). No significant difference in mean glucose levels was observed between patients of the 2 groups who used insulin (CG: 106·83 (7·45) vs EG: 109·83 (9·04) mg/dL; *P*=0·342). No difference in the frequency of cesarean section was observed between groups (n: 21 of 32 in EG vs n 24 of 32 in CG; *P*=0·412). 3 cases of preterm delivery in each group (gestational age at birth ranging from 35 to 36 weeks).
**4**	Kolivand et al., 2019 [47]	Minimal risk	New **self-care guide package** plus three face-to-face **educational sessions**	**Routine clinical services**: physician visits and short training regarding nutrition, blood glucose control, and insulin injections	Superior effects on **maternal self-efficacy** and 2-h **postprandial plasma glucose**, **Apgar scores**, and **neonatal hospitalization**. The mean (±SD) self-efficacy score was higher in the intervention than control group (74·4 ± 7·0 vs 36·4 ± 5·2, respectively; *P* < 0·001). Mean 2-h postprandial plasma glucose was lower in the intervention than control group (105·1 ± 17·6 vs 127·2 ± 20·4 mg/dL, respectively; *P* < 0·001). Newborn hospitalization rate was higher in the control group (*P* < 0·001).	–	No significant difference in mean **fasting plasma glucose** between the two groups (*P* = 0·163) No significant differences in the **weight and age of the neonate at delivery** or in the **type of delivery** between the two groups.
**5**	Kokic et al., [31]	Minimal risk	Experimental group was treated with standard antenatal care for gestational diabetes mellitus, and **regular supervised exercise programme** plus daily brisk walks of at least 30 minutes. The exercise programme was started from the time of diagnosis of diabetes until birth. It was performed two times per week and sessions lasted 50–55 min.	Control group received only **standard antenatal care** for gestational diabetes mellitus.	The experimental group had lower postprandial glucose levels at the end of pregnancy (*P* < 0·001).	Neonatal body mass index was higher in the experimental group (*P* = 0·035).	No significant difference between groups in the level of fasting glucose at the end of pregnancy. No significant differences in the rate of complications during pregnancy and birth, need for pharmacological therapy, maternal body mass and body fat percentage gains during pregnancy, and neonatal Apgar scores, body mass and ponderal index.
**6**	Tian et al., 2021 [53]	Moderate risk	**Health education and lifestyle management delivered through a WeChat group** chat Participants received WeChat group management in addition to standard clinical prenatal care. Participants received management on a weekly basis. In particular, every Monday, researchers would issue a briefing to encourage patients to take an active part in the control of their GDM and a task card to pinpoint the basic requirements, including diet advice, examples of meals from other group members, and exercise rules. Patients performed self-management according to the basic criteria provided for their actual situation and shared photos of their meals and snacks, daily exercise, and experience regarding BG control. Researchers would give individualized guidance for self-management or use a group member’s situation as an example for others. On weekends, the researchers prepared lessons and articles for group members to learn different aspects of pregnancy and GDM, including rudimentary knowledge, disease management, psychology, and past cases. We encouraged the sharing of learning experiences and notes in the form of peer interactions and support groups. If there were any questions regarding the project, pregnancy, or GDM, patients could seek answers from the group chat. This weekly management continued until delivery.	**Standard clinical prenatal care** Participants were taught basic information about GDM and how to do self-management, including how to conduct blood glucose monitoring, what the target BG values are, and how to keep a lifestyle diary.	Additional instant messaging platforms, such as WeChat, used for health education and lifestyle intervention in China tend to be more effective for blood glucose control than standard clinical prenatal care alone. The glycemic qualification rate of the intervention group was higher than that of the control group at nearly all time points in Groups 1 to 3, among which 3 time points reached statistical significance: Group 1 at T3 (54·8% vs 83·3%) and Group 2 at T3 (62·5% vs 80·0%) and T7 (75·0% vs 100%).	–	None of the pregnancy outcomes measured, including delivery mode, premature rupture of the membranes, preterm birth, infant's birth weight, and postpartum hemorrhage, were significantly different between the control and intervention groups.
**7**	Barati et al., 2021 [54]	Moderate risk	**30 grams of oat bran daily for 4 weeks at lunch and dinne**r PLUS diet for gestational diabetes.	**Routine diet for gestational diabetes**.	The addition of oat bran to the standard diet for pregnant women with gestational diabetes reduced fasting blood glucose and two-hour postprandial glucose. Mean fasting blood glucose and two-hour postprandial glucose decreased significantly in the intervention group compared with the control group (*P* < 0·001). Two weeks after the start of oat bran consumption, the mean two-hour postprandial glucose was 122·17 (3·91) in the control group and 115·37 (3·14) in the oat bran group, which was significantly different (*p* < 0·001). Four weeks after start of the consumption of oat bran, the mean two-hour postprandial glucose was 117·49 (11·34) in the control group and 104·04 (5·48) in the oat bran group, which was significantly different (*P* < 0·001).	–	The two groups were not significantly different in terms of average fat intake (*p* = 0·67), average carbohydrate intake (*p* = 0·28), protein intake (*p* = 0·23) and fibre intake (*p* = 0·46).
**8**	Mohebbi et al., 2019 [32]	Moderate risk	**Self-management education programme** was presented in four sessions lasting 35-40 minutes for each during a month. Phone calls as small booster were conducted which served as a quick reference to education and reminder to study participants. Content of educational programs included basic information regarding GDM facts, figures and self-management based on HBM constructs like perceived susceptibility and severity of gestational diabetes, barriers and benefits of self-management and perceived self-efficacy and self-management incorporating cues to actions. Strategies such as setting achievable goals and use of motivational interviewing to increase self-efficacy were also used in educational sessions. Ways of social support from family were considered offering empathy, concern, encouragement, or caring to the women. Moreover, self- monitoring of blood glucose was used as a way to teach participants about their disease using pictures and simple instructions. At the end of each session, the educator reviewed the important topics of the session and women were encouraged to ask their questions which were answered accordingly.	**Routine** clinic-based education.	There were significant (*P*<0·001) improvements in the self-management education programme group compared to the control group at 3 and 6 months after the intervention in all outcomes including HbA1c, self-management, self-efficacy, cues to action, perceived benefits, perceived barriers, perceived severity, perceived susceptibility	–	–
**9**	Al Hashmi, 2018 [48]	Moderate risk	**Self-efficacy-enhancing intervention** (SEEI) PLUS standard antenatal care. The SEEI group received an additional individualised health education intervention utilising different self-efficacy-enhancing strategies (i.e. motivational messages, role modelling, goal-setting and mastery experience) designed to encourage women to maintain recommended healthy behaviours. First, the participants watched an educational video designed solely for the purposes of the study. The video focused on general information about GDM and GDM-related maternal and neonatal complications, as well as information about the importance of healthy lifestyle behaviours— such as a healthy diet, exercise and maintaining self-monitored BG levels to prevent GDM complications— and measures to prevent post-partum T2DM. The physical activities recommended in the video conformed with standard cultural beliefs and religious practices in Oman by focusing on safe exercises which could be conducted indoors and in private (i.e. walking, swimming and dancing). After watching the video, participants in the SEEI group were encouraged to practice the recommended activities during the session. The participants were provided with a BG metre and were trained to check and record their BG levels; they were requested to do this four times per day during the study period. In order to enhance adherence to the recommended healthy behaviours, the participants were encouraged to write down specific and measurable goals. A pamphlet summarising the content of the educational session was distributed to all participants before the end of the session. In addition, they received short biweekly motivational text messages for four weeks to reinforce the information given during the educational session. Finally, a refresher session was given at 32–35 gestational weeks via telephone.	**Standard antenatal care**, including routine antenatal visits, monthly blood sugar profiles, fasting blood sugar testing at every visit, glucose monitoring at home and individualised educational sessions with a diabetes dietician.	The SEEI was found to significantly improve perceived self-efficacy and actual adherence to healthy behaviours among a group of Omani women with GDM. There was a significant positive difference between the SEEI and control groups in terms of pre-post change in scores for both perceived self-efficacy: 9·9 (19·6) versus −1·8 (17·6); *P* <0·05 and actual adherence to healthy behaviours – diet, exercise, BG monitoring: 1·5 (1·1) versus 0·4 (0·8); *P* <0·01.	–	–
**10**	Bartholomew et al., 2015 [35]	Moderate risk	The use of **cell phone–Internet technology (CIT) for self-management** (monitoring) of hyperglycemia during pregnancy 3-hour diabetes education class taught by certified diabetes educators. Women who required medication (insulin or glyburide) were provided personalized instruction regarding correct usage. All women received equivalent **education, training, and consultation regarding a carbohydrate-controlled diet, exercise, SMBG, and reporting SMBG results**. All women received the same glucose meters (OneTouch; LifeScan, Inc., Milpitas, Calif.) and testing supplies. They were instructed to perform SMBG four times per day (fasting and 2 hours postprandially) and record values using the reporting method to which they were assigned. PLUS Women using the CIT method were advised to upload their blood glucose results at least weekly, although they could upload at every test, every day, or at their convenience within that timeframe. The system uploaded every value in the meter each time an upload occurred. Uploading began by turning on the cell phone and glucose meter. The wireless device was plugged in to the glucose meter and turned on. The phone was placed within 3 feet of the wireless device. Participants pressed a menu button and then selected the “collect” option on the phone menu to start the application. A confirmation of data receipt was displayed on the phone. Supplementary Figure 1 shows the components of the CIT glucose meter system. Each week, MFM physicians reviewed the blood glucose values on the Web site. The nurses communicated the recommendations to patients by telephone. Those using the CIT method could review their progress on the Web site or in graphs created on the phone. They also received automatic encouraging text messages. Sample text messages were: • “You didn’t submit readings for the second week in a row. Try to submit your readings every week.” • “Did you notice your overall glucose average rose over the past week?” • “Thanks for submitting your readings. Keep up the good work!” CIT technical support was available by telephone 12 hours/day.	All women received equivalent **education, training, and consultation regarding a carbohydrate-controlled diet, exercise, SMBG, and reporting SMBG results.** 3-hour diabetes education class taught by certified diabetes educators. Women who required medication (insulin or glyburide) were provided personalized instruction regarding correct usage. All women received the same glucose meters (OneTouch; LifeScan, Inc., Milpitas, Calif.) and testing supplies. They were instructed to perform SMBG four times per day (fasting and 2 hours postprandially) and record values using the reporting method to which they were assigned. PLUS Women using the control method were advised to record blood glucose values in a log book and report their handwritten glucose results to the program nurse each week by dictating the values on the voicemail system. Nurses listened to the voicemail messages and recorded the values on paper. MFM physicians reviewed the paper records weekly to make recommendations. Nurses then communicated the recommendations to the women by telephone.	Compliance with SMBG reporting was higher during use of the CIT method for total, fasting, and 2-hour postprandial glucose values. The mean 2-hour postprandial SMBG value was 108·3 mg/dL when the CIT method was used first and 112·7 mg/dL when the control method was used first (*P* = 0·023). The mean fasting blood glucose value was 89·5 mg/dL when CIT was used first and 92·5 mg/dL when voicemail was used first (*P* = 0·049). With regard to the secondary outcome of satisfaction, 68·9% of women preferred (“liked best”) the CIT method compared to 24·3% who said they preferred (“liked best”) the voicemail method (*P* <0·001); More than half (59·5%) of the women found the automatic text messages to be “always helpful,” whereas 24·3% found them “often helpful,” 10·8% found them “rarely helpful,” and 5·4% found them “never helpful.”	–	–
**11**	Asemi et al., 2014 [40]	Moderate risk	**Dietary Approaches to Stop Hypertension** (DASH) eating plan. The DASH diet was rich in fruits, vegetables, whole grains, and low-fat dairy products, and contained lower amounts of saturated fats, cholesterol, and refined grains with a total of 2400mg/day sodium.	The **control diet** was designed to contain 45–55% carbohydrates, 15–20% protein and 25–30% total fat.	Consumption of DASH diet for 4 weeks among pregnant women with GDM resulted in improved pregnancy outcomes. 46·2% of women in the DASH diet needed to have a caesarean section, this percentage for the control group was 80·8% (*P*= 0·01). The percentage of those who needed to commence insulin therapy after intervention was also significantly different between the two groups (23% for DASH vs 73% for control group, *P*<0·0001). Consumption of the DASH diet led to a significant reduction in the birth of macrosomic infants compared with the control diet (3·8 vs 38·5%, *P*=0·002). Infants born to mothers on the DASH diet had significantly lower weight (3222·7 vs 3818·8 g, *P*<0·0001), head circumference (34·2 vs 35·1 cm, *P*= 0·01) and ponderal index (2·50 vs 2·87 kg/m3, *P*<0·0001) compared with those born to mothers on the control diet.	–	No significant difference in mean gestational age was found when comparing the DASH and control diets. Prevalence of polyhydramnios was not significantly different between the two groups. No significant difference in mean length and Apgar score of the newborns when comparing the DASH and control diets.
**12**	Grant et al., [41]	Moderate risk	**Low-glycaemic-index (LGI) diet**	**Routine diabetic diet**	More postprandial glucose values were within target on low-GI (58·4% of *n*=1891) than control (48·7% of *n*=1834; *p*<0·001).	–	Glycaemic control improved on both diets.
**13**	Mendelson et al., 2008 [45]	Moderate risk	**Supplementary 1-hour education session for diabetes education** PLUS usual obstetric care reinforcement by a Parish Nurse.	**Usual obstetric care**	Significantly improved Health Promoting Lifestyle Profile II scores (self-reported health promoting behaviors) in the Parish Nurse Intervention Program group post-intervention compared with usual obstetric group.	–	No significant differences between groups regarding glycemic control, macrosomia, or days of maternal or neonatal hospitalization were found.
**14**	Daniel et al., 2014 [29]	High risk	8 weeks **aerobic dance exercise** consisting of three exercise sessions per week, 40 minutes per session for the first 4 weeks and 60 minutes per session for the last 4 weeks.	**Routine care and activities of daily living**	Significant improvement in the fasting blood sugar (*p*= 0·001) of the exercise group.	–	–
**15**	Hu et al., 2014 [38]	High risk	**Low glycaemic index diet**	**Routine diabetic control diet**	A low-GI staple diet significantly reduces postprandial glucose levels Glucose levels were significantly reduced in the low-GI staple diet group (all *P* < 0·01) and the control group (all *P* < 0·008). Postintervention glucose values after breakfast, lunch, and dinner were significantly reduced in the treatment group compared with those in the control group (all *P* < 0·05). The percentage changes from baseline of all glucose values were significantly greater in the treatment group than in the control group (all *P* < 0·05).	–	–
**16**	Ural & Beji, 2021 [46]	High risk	**Health-Promoting Lifestyle Education Programme** and usual care	**Usual care**	Improvement in the healthy lifestyle behaviours and quality of life in the intervention group. The rates of macrosomia were low for the neonates in the intervention group.	–	–
**17**	Yang et al., 2018 [33]	High risk	WeChat platform-based using both a smartphone-based telemedicine system and articles providing continuous **health education** PLUS routine outpatient treatment and health education guidance	**Routine** outpatient treatment and health education guidance	Fasting blood glucose (FBG) and 2-h postprandial blood glucose (PBG) were significantly lower and premature delivery was significantly less likely in intervention group than in control group (all *P* < 0·05). Pregnancy-induced hypertension had a higher incidence in control group (*P* <0·05)	Compared with control group, caesarean section was more likely in intervention group (*P* < 0·05).	–
**18**	Ibrahim & Saber, 2019 [34]	High risk	4 educational sessions each one lasted for 30 minutes included lectures, PowerPoint, and group discussion. The **educational programme** involved notes on the general knowledge of gestational diabetes including definition, aetiology, high-risk groups, clinical manifestation, maternal and foetal complications, diagnosis, management, self-care practice such as following a dietary regimen, physical exercise, drug regimen with insulin, and postnatal management. Health education were for the women, and their families. Modules for education included power point, lectures, and brochures that contained pictures for self-measuring of random blood glucose level, dietary recommendations to maintain blood sugar within the normal range, drug regimen.	**Routine** pre-natal care	Statistically significantly higher proportions of women had satisfactory knowledge in the study group compared to the control group. Significantly more women were satisfied with their knowledge about gestational diabetes after the intervention in the study group was than in the control group. Significantly higher proportions of favourable practices were found in the study group compared to the control group. Significantly more women had more total favourable self-care practices after the intervention in the study group than in the control group. Statistically significantly higher proportions of complications in the control group than the study group were found. The rate of normal delivery was higher in the study group, and the rate of caesarean section was higher in the control group. The rates of foetal health problems were significantly higher in the control group than the study group e.g., jaundice, macrosomia etc.	–	–
**19**	^b^Yu et al., 2014 [36]	High risk	**Continuous glucose monitoring** (CGM) PLUS standard antenatal care. Visits included downloads and analysis of data in meter and sensor (only for patients in CGM group), nutrition consultation, education of information on blood glucose testing and self-care activities, and getting an individualized diabetes care prescription, which was arranged by the same obstetric diabetes team.	**Standard antenatal care** using intermittent SMBG test from capillary blood obtained by the finger prick technique. Visits included nutrition consultation, education of information on blood glucose testing and self-care activities, and getting an individualized diabetes care prescription, which was arranged by the same obstetric diabetes team.	Better glycaemic control and improved pregnancy outcomes in the CGM group by reducing the risk of pre-eclampsia and caesarean delivery, decreasing the birth weight, and improving neonatal complications.	–	–
**20**	^c^Sunsaneevithayakul et al., 2004 [37]	High risk	^b^Prescribed **intensive diet therapy for 3 days**. received extensive dietary counseling by a well-trained diabetes nurse educator and physicians. The diabetic counseling and teaching, as well as the obstetric management, were done during admission.	^b^**Standard treatment** of all subjects involved diabetes education, control of hyperglycemia, with fetal and maternal surveillance. Daily caloric assignment was calculated based on ideal body weight, 30-35 Kcal/kg.	Short course of intensive dietary therapy during the 3 days of admission enabled good glycemic control such that 57·4% did not require insulin therapy.	–	–
**21**	Artal et al., 2007 [42]	High risk	**Exercise and diet**. Exercise was equivalent to a 60% symptom-limited VO2 max.	**Diet alone**	Maternal weight gain per week was significantly lower in the exercise and diet group.	–	Other pregnancy and foetal outcomes such as complications, gestational age at delivery, and rate of caesarean delivery were similar in both groups.
**22**^a^	^**a**^Kgosidialwa, et al., 2015 [43]	High risk	^**a**^**Diet and exercise**. Each patient received an hour-long, individual consultation with a dietician at time of GDM diagnosis and additional consultations were arranged if deemed necessary. Exercise was tailored to the individual woman basing on the American Congress of Obstetrics and Gynaecology. In addition, women had access to a phone service to contact the midwife/diabetes nurse specialist during office hours for advice. Women were advised to monitor their blood glucose levels using a glucometer (capillary glucose monitoring) at least 7 times a day (premeal, 1-hour post meals, and at bedtime). Blood glucose targets were set at 5·3 mmol/L for fasting/premeal glucose levels and 7·8 mmol/L 1 hour post meals.	^**a**^**Women with normal glucose tolerance** – received **routine** antenatal care	^**a**^LGA and macrosomia rates were lower in the MNT and exercise treated GDM group compared with the NGT group.	–	All other adverse outcomes were similar between groups.
**23**	^a,^^d^Wang et al., 2015 [44]	High risk	^d^**Exercise intervention**. Exercise intervention means sit less, take more steps, be more active, incorporate light and moderate physical activity as much as possible into their daily life et al., and diet intervention means reduce intake of sugar, eat more vegetables, reduce fat intake, and the total energy intake 1800 calories a day in all.	^d^**Women without GDM** PLUS **women with GDM without exercise intervention**	Women with GDM with exercise intervention (GDM-E) had the lowest BMI increase during late and mid-pregnancy than women with GDM without exercise intervention (GDM-nE) (2·05 ± 1·32 kg/m^2^ vs. 2·40 ± 1·30 kg/m^2^, *p* < 0·01) and non-GDM women (2·05 ± 1·32 kg/m^2^ vs 2·77 ± 1·21 kg/m^2^, *p* < 0·01). Moreover, GDM-E group experienced a significantly lower risk of preterm birth (5·58 % vs. 7·98 %, *p* < 0·001), low birth weight (1·03 % vs. 2·06 %, *p* < 0·001) and macrosomia (9·51 % vs. 11·18 %, *p* < 0·05) than GDM-nE group. Women with GDM with both dietary and exercise intervention had the lowest rate of macrosomia.	–	–
**24**	^**a**^ Shi et al., 2016 [49]	High risk	Pregnant women with GDM were routinely advised to receive **MNT counselling** where trained nutritionists provide individualized MNT programs for pregnant women with confirmed GDM. They also established daily energy requirements and calorie supply proportions of the three major nutrients in accordance with the China Medical Nutrition Therapy Guideline for Diabetes (2010) based on the pre-pregnancy body type, gestational age at the time of GDM diagnosis, increase in body weight during pregnancy, blood pressure, and lipid outcomes. They then provided suggestions with regard to the type of food, specifically quantifying the recommended intake for each type of food. They also assisted in the selection of foods among similar food types via the “method of food exchange serving” to diversify the patients' diets while ensuring a balanced intake of all necessary nutrients. Finally, they suggested reasonably arranged meal times and foods in each meal based on blood glucose monitoring data, recommended staple foods with low glycemic index values, and emphasized eating many small meals to reduce each meal's glycemic load. Regular postprandial exercise was also recommended. Pregnant women were encouraged to obtain private fast blood glucose meters, kitchen scales, and body weight scales for self-monitoring of finger-prick blood glucose, food intake, and body weight at home.	**No MNT**	The fasting plasma glucose, 2-hour blood glucose, and weight gain at 28 weeks, 32 weeks, and 36 weeks as well as intrapartum were lower in the MNT group than in the non-MNT group. Total weight gain during pregnancy and the rates of adverse events during pregnancy were lower in the MNT group compared to the non-MNT group (all *p* < 0.05). Moreover, 92·2% of the participants in the MNT group had a normal oral glucose tolerance test result, and the rate of exclusive breastfeeding within 4 months after delivery was 54·4% in the MNT group; both were higher than those of the non-MNT group (66·3%, *p* < 0·001; 29·3%, *p* < 0·05).	–	–
**25**	Perichart-Pereraet al., 2009 [50]	High risk	Intensive **MNT programme** The MNT program consisted of individual nutrition counseling with an intensive education component performed by one clinical dietitian. The program included nutrition assessment, nutrition intervention, and capillary glucose self-monitoring. Specific materials were designed for nutrition therapy and self-monitoring education. Nutrition recommendations were based on nutrition practice guidelines for gestational diabetes developed and published by the American Dietetic Association. Women received a glucose meter (Optium MediSense, Abbott Laboratories, Bedford, MA) and strips to perform capillary blood glucose self-monitoring 2 days a week, 6 times a day (before and 2 hours after each meal). Fasting and 2 hours postprandial serum glucose was also measured every 2 weeks by a glucose oxidase method. Until the end of pregnancy, all women received follow-up every 2 weeks by the dietitian and the endocrinologist, who was responsible for prescribing insulin, as needed, to meet glycaemic goals.	**Routine antenatal care** in a historical control. Usual routine care in the control group included monthly medical visits with the endocrinologist before 28 weeks of gestation, and every 2 weeks thereafter. Most women attended 1 initial nutrition orientation group session where they received dietary information from a technician. Less than 5% of them had a glucose meter to perform capillary glucose self-monitoring	Serum 2 hours postprandial glucose values during the last visit tended to be lower in women in the MNT programme compared with women in the control group (107·05 ± 23·83 vs 115·64 ± 36·11). The number of total perinatal complications was higher in the control group than the MNT programme (P = 0·005). Fewer women in the MNT programme (27·3%) had ≥1 perinatal complications, than the control group (45·3%, P = 0·013). Fewer women developed preeclampsia in the MNT programme than the control group (2·3% vs 16·3%; P = 0·001). First maternal hospitalization (due to uncontrolled hyperglycemia) was less frequent in the MNT programme (5·7% vs 62·8%; *P* < 0·001). Moreover, women in the MNT programme did not require a second hospitalization. No neonatal deaths and lower NICU admissions were also observed in the MNT programme (P = 0·001).	–	Among women with gestational diabetes, more women in the control group used insulin than women in the MNT programme (56·4% vs 35·9%) but the doses prescribed were not statistically different (16 vs 0 unit/day, P = 0·052). Fasting glucose values were not different between women in the MNT programme and the women in the control group. Although there were no statistically significant differences, a greater proportion of women in the control group had elevated values of serum fasting and 2 hours postprandial glucose levels (fasting: 37·2% vs 33·0%, P = 0·715; 2 hours postprandial: 37·2% vs 26·1%, P = 0·169). Although the frequency of prematurity, macrosomia, and low birth weight were not statistically different among the groups, the MNT programme showed lower rates. Intrauterine death was similar between the 2 groups.
**26**	Murphy et al., 2004 [51]	High risk	**Nutrition counselling** for patients with gestational diabetes mellitus (GDM) **in small group**. Nutrition counselling, provided by a registered dietitian, consisted of a 1-hour interactive education session using a tabletop flip chart. Supporting written materials were used in both categories to reinforce the topics discussed. Subjects completed a knowledge assessment test based on the content of the counselling session, which consisted of 12 multiple choice questions, at 3 time points: prior to nutrition counselling, immediately after counselling and 1 week after counselling.	**Nutrition counselling** for patients with gestational diabetes mellitus (GDM) in **individual counselling**. Nutrition counselling, provided by a registered dietitian, consisted of a 1-hour interactive education session using a tabletop flip chart. Supporting written materials were used in both categories to reinforce the topics discussed. Subjects completed a knowledge assessment test based on the content of the counselling session, which consisted of 12 multiple-choice questions, at 3 time points: prior to nutrition counselling, immediately after counselling and 1 week after counselling.	A total of 27 dietitian hours were saved with small-group counselling. Women with GDM can be effectively and cost-efficiently counselled on nutrition in small-group settings.	–	Post counselling results showed a significant improvement in knowledge, regardless of counselling method (*p*<0·0001). Post counselling results showed no difference in knowledge improvement between participants in small-group counselling and those who received individual counselling, based on equivalence testing (95% confidence interval [CI]: -3·7 to 5·5). One-week follow-up results demonstrated that knowledge was retained in both counselling categories (95% CI: -6·2 to 2·4).
^**c**^**27**	^c^Hayashi et al., 2018 [4]	High risk	^c^**Daily walking** for GDM management. The total amount of daily walking was estimated from the number of steps taken and the amount of exercise performed daily, as measured with an accelerometer Participants attached the accelerometer to the waistbands of their skirts or pants, as instructed at the time of recruitment by investigators. The accelerometers assessed daily walking for a total of 7–12 weeks because periodic pregnancy examinations were performed every 4 weeks on the basis of the number of steps taken and the amount of exercise performed every day from the second trimester to the third trimester. The accelerometers were removed during sleeping and bathing. In the third trimester, the participants removed the accelerometers permanently and completed questionnaires that assessed dietary intake.	^c^No control group	^c^Simple walking for light intensity physical activity is effective for controlling the CGL in pregnant women with GDM. ^c^There was a significant negative correlation (r = −0·603, P = 0·014) between the post- to pre-research casual glucose level (CGL) ratio and the number of steps walked daily. When the study was completed, the 11 participants who walked ≥6000 steps/day showed significantly lower CGL (95 + 10 mg/dL [mean + SD]) than the 13 participants in the <6000 steps/day group (111 + 18 mg/dL) (P = 0·013).		^c^No significant correlation (r = −0·004, P = 0·986) was detected between the ratio of hemoglobin A1c and the number of steps taken.

*LGI diet* Low–glycaemic index diet, *GI* Glycaemic index, *HF* High fibre, *EG* Exercise group, *CG* Control group, *BG* Blood glucose, *CIT* Cell phone–Internet Technology, *SMBG* Self-monitoring of blood glucose, *GDM* Gestational diabetes mellitus, *HBM* Health belief model, *HbA1c* Glycated haemoglobin, *CGM* Continuous glucose monitoring

*MNT* Medical nutrition therapy, *LGA* Large for gestational age, *NGT* Normal glucose tolerance, *CGL* Casual glucose level

^a^retrospective cohort study with control

^b^prospective cohort study with control

^c^longitudinal study [4] or case series [37] without control group, participants served as their own controls

^d^not a proper control group because experimental and control groups were different populations

#### Summary of individual study outcomes

Majority (25) of the studies showed that community-based non-pharmacological interventions were more effective than control interventions which included routine/standard prenatal care, placebo, or no treatment in improving the greatest number of maternal and new-born outcomes. 15 studies reported similar effectiveness in comparatively fewer number of outcomes in the intervention and control groups. Only one study reported a superior improvement in a neonatal outcome (neonatal body mass index), and only one study reported a superior improvement in a maternal outcome (reduced caesarean section use), both when compared with control (routine/standard prenatal care).

#### Summary of community-based non-pharmacological interventions

##### Self-management programmes

Nine studies investigated self-management programmes which involved face-to-face educational sessions and self-care guide package [47], health education and lifestyle management delivered using social media (WeChat) plus motivational briefing to promote self-care in relation to diet, exercise, and blood glucose control [53], self-management education programme involving goal setting, motivational interviewing to increase self-efficacy, social support, self-monitoring of blood glucose, plus phone call reminder of education [32], self-efficacy enhancing intervention involving individualised health education comprising motivational messages, role modelling, goal-setting and mastery experience to facilitate healthy behaviours [48], cell phone–internet technology (CIT) for self-management (monitoring) of hyperglycemia during pregnancy [35], supplementary 1-hour education session for diabetes education [45], health-promoting lifestyle education programme [46], continuous health education using a smartphone-based telemedicine system [33], group educational programme enhancing health-related knowledge and facilitating self-care [34].

##### Medical nutrition/diet therapy interventions

Nine studies examined medical nutrition/diet therapy programmes which involved low–glycaemic index (LGI) diet [38, 39, 41], oat bran plus routine diet for gestational diabetes [54], dietary approaches to stop hypertension (DASH) diet that is rich in fruits, vegetables, whole grains and low-fat diary products [40], 3-day intensive diet therapy [37], medical nutrition therapy counselling [49–51].

##### Exercise/physical activity programmes

Five studies investigated exercise/physical activity programmes which involved resistance exercise [30], regular supervised exercise plus daily brisk walking [31], aerobic dance exercise [29], physical activity lifestyle programme [44], and daily walking programme [4].

##### Combined diet and exercise interventions

Two studies investigated combined exercise and diet therapy programmes which involved exercise equivalent to a 60% symptom-limited VO2 max and diabetic diet [42], and consultation with a dietician plus individually tailored exercise [43].

##### Calcium and vitamin D supplement therapy

One study examined the use of combined 1000 mg of Calcium and two pearls containing 1250 μg (50 000 IU) of cholecalciferol [52].

##### Continuous glucose monitoring intervention

One study investigated the use of continuous glucose monitoring which involved the use of continuous glucose monitoring system to assess 24-hour glucose fluctuations (every 10 seconds and an average value stored every 5 minutes. This provides up to 288 measurements per day, and offers a complete view of glucose profile about the direction, magnitude, duration, frequency, and causes of fluctuations in blood glucose levels [36].

### Results of syntheses

#### Effectiveness of community-based non-pharmacological interventions

Meta-analysis was precluded due to different intervention content in experimental and control groups, study designs, and outcomes (Tables 2 and 4).

##### Effectiveness of self-management programmes

Self-management programmes consistently improved most health behaviour-related outcomes such as self-efficacy [32, 47, 48], lifestyle behaviours [46, 48], self-management behaviour [32], self-monitoring of blood glucose [35], adherence to recommended healthy health promoting behaviours [45], cues to action [32], perceived benefits [32], perceived barriers [32], perceived severity [32], perceived susceptibility [32], and satisfactory knowledge [34] better than routine obstetric care.

Two-hour postprandial blood glucose was also consistently better improved by self-management programmes than routine care [33, 35, 47].

Only one clinical trial each measured quality of life [46] and satisfaction [35], pregnancy-induced hypertension [33] with all reporting better improvements in the self-management programmes.

No differences between self-management programmes and routine care were found for infant birth weight [47, 53] and age of neonate at delivery [47], premature rupture of membranes [53], postpartum haemorrhage [53], days of maternal/neonatal hospitalisation [45].

Other maternal and neonatal clinical outcomes were conflicting. Two clinical trials (one cross-over trial) reported no difference in fasting blood glucose in the routine care and self-management programmes [35, 47]; but one non-RCT reported superior improvements in fasting blood glucose in the self-management programmes than routine care [33]. One study each reported superior effects of self-management programmes on blood glucose control (glycaemic qualification rate) [53], glycated haemoglobin [32], but another study reported no significant differences in glycaemic control [45] compared with routine care. No differences were found in macrosomia [45] in one study whereas another study reported lower rates of macrosomia in the self-management programmes compared with routine care [46]. One study reported no differences in preterm birth [53] but another study showed lower rates of premature delivery in the self-management programme compared with routine care [33]. Two studies reported no differences in the type of delivery [47, 53], whereas one study reported that caesarean section was less in the routine care group compared with self-management [33].

##### Effectiveness of medical nutrition/diet therapy interventions

Medical nutrition/diet therapy interventions consistently improved postprandial blood glucose levels more than routine diabetic diet [38, 41, 49, 50, 54].

One study showed that small group nutritional counselling saved 27 dietitian hours compared to individual nutritional counselling, with knowledge levels being similar in both treatment groups [51].

Fasting blood glucose was better improved by the addition of 30 grams oat bran daily to routine diabetic diet for 4 weeks than routine diabetic diet alone [54]. Superior improvements in fasting blood glucose was produced by a medical nutrition therapy (MNT) counselling compared with a no MNT group [49]. Another MNT programme produced similar improvements in fasting blood glucose as routine obstetric care [50].

Similar glycaemic control was observed between a low glycaemic index diet and a routine diabetic diet consisting of conventional high fibre diet [41]. However, a three-day intensive diet therapy produced superior glycaemic control than routine diabetic care such that fewer people required insulin therapy [37].

Similar outcomes between a low glycaemic index and a conventional high fibre diet were produced for infant birth weight, ponderal index (within normal), maternal weight, foetal abdominal circumference [54]. Likewise, similar outcomes were produced by an intensive MNT programme and routine obstetric care in insulin use, prematurity, macrosomia, low birth weight, and intrauterine death [50]. MNT counselling produced superior outcomes in maternal weight, rates of adverse events, and exclusive breast feeding than a no MNT control [49]. Similarly, an intensive MNT programme produced better outcomes in rates of perinatal complications, preeclampsia, maternal hospitalisations, neonatal intensive care unit admissions, and had no neonatal deaths compared with routine obstetric care [50]. Dietary approaches to stop hypertension (DASH) diet that is rich in fruits, vegetables, whole grains and low-fat diary products produced superior pregnancy outcomes (lower caesarean delivery and insulin therapy), lower rates of macrosomia, lower birth weight and head circumference, and lower ponderal index; but similar outcomes in gestational age, polyhydramnios, newborn length, and Apgar score compared to a control diet (45-55% carbohydrate, 15-20% protein, 25-30% total fat) given as routine care [40].

##### Effectiveness of exercise/physical activity programmes

Postprandial blood glucose levels were better improved by regular supervised exercise plus daily brisk walks than routine obstetric care [31]. Similarly, postprandial blood glucose levels were improved by a daily walking intervention in a pre-post-test study with no control group [4]. The effects of exercise/physical activity programmes were mostly inconsistent for other outcomes.

Fasting blood glucose was better improved by an aerobic dance exercise intervention than routine obstetric care [29]. In contrast, a regular supervised exercise plus daily brisk walking intervention and routine obstetric care produced similar improvements in fasting blood glucose [31]. Resistance exercise produced greater improvements in glycaemic control (weeks within target blood glucose range) than routine obstetric care but resistance exercise and routine obstetric care produced similar mean glucose levels [30].

Resistance exercise produced greater reduction in people requiring insulin, new-born birthweight but similar outcomes in quantity of insulin required by patients, time interval between patient inclusion in study and insulin use, rates of caesarean section, rates of preterm when compared with routine obstetric care [30]. Physical activity lifestyle programme produced better improvements in maternal body mass index, rates of preterm birth, rates of low birth weight, and macrosomia than a control group without such intervention [44]. Regular supervised exercise plus daily brisk walks produced similar outcomes as routine obstetric care on rates of pregnancy and birth complications, need for pharmacological therapy, maternal body mass and body fat percentage, neonatal Apgar scores, ponderal index. However, neonatal body mass index was better in the routine obstetric care group than the exercise/physical activity group [31].

##### Effectiveness of combined diet and exercise interventions

Diet and exercise was superior to diet alone in reducing maternal weight gain per week but similar outcomes were observed for other pregnancy and foetal outcomes including complications, gestational age at delivery, and rate of caesarean delivery [42]. Diet and exercise were better in improving the rates of large for gestational age and macrosomia compared with pregnant women with normal glucose tolerance placed on routine obstetric care, although other adverse outcomes were similar between the two groups [43].

##### Effectiveness of calcium and vitamin D supplement therapy

Significant decrease in the rates of caesarean delivery, maternal hospitalisation, hyperbilirubinaemia, newborn hospitalisation were observed with combined 1000 mg of Calcium and 1250 μg of cholecalciferol compared with placebo. No case of macrosomia was seen with the vitamin supplementation, in contrast to placebo [52].

##### Effectiveness of continuous glucose monitoring intervention

Better glycaemic control, reduced risk of pre-eclampsia and caesarean delivery, and reduced birth weight and neonatal complications were produced with continuous glucose monitoring plus routine obstetric care compared to routine obstetric care alone [36].

### Reporting bias in overall results

There were no systematic differences in the consistent and inconsistent outcomes based on study rating of bias, study design and the size of individual studies.

### Certainty of overall results

Strong evidence showed that the community-based self-management programmes improved self-efficacy [32, 47, 48], and 2-hour postprandial blood glucose [33, 35, 47], better than routine care. Moderate evidence suggests that the self-management programmes improved life style behaviours [46, 48], better than routine care. Moderate evidence indicated that the self-management programmes produced similar outcomes on infant birth weight as routine care [47, 53].

The strength of evidence for the effectiveness of the self-management programmes for some outcomes was conflicting. Moderate evidence suggests that the self-management programmes produced similar outcomes as routine care for fasting blood glucose [35, 47] but very low evidence suggest that they are superior to routine care [33] for fasting blood glucose. There is very low evidence that the self-management programmes were superior or as effective as routine care in improving blood glucose control [32, 45], macrosomia [45, 46], and preterm delivery [33, 53]. There is moderate evidence that the self-management programmes produced similar outcomes as routine care in reducing the rates of caesarean delivery [47, 53], but very low strength of evidence that they are inferior to routine care [33] in reducing the rates of caesarean delivery.

There is moderate evidence that community-based medical nutrition/diet therapy interventions were more effective than usual care in improving postprandial blood glucose levels [38, 41, 49, 50, 54].

The strength of evidence for the other outcomes and the effectiveness of the other community-based non-pharmacological interventions was uncertain because only one study examined them.

## Discussion

This systematic review of community-based non-pharmacological interventions for pregnant women with gestational diabetes mellitus indicate that these interventions were more effective than routine care in improving health behaviour related outcomes and two-hour postprandial blood glucose. Other outcomes of these programmes were less consistent. Community-based self-management programmes were superior to or as effective as routine care in improving fasting blood glucose, blood glucose control, glycated haemoglobin, macrosomia/infant birth weight, and preterm delivery. There was a trend towards community-based self-management programmes being superior to routine care in improving self-management behaviour, self-monitoring of blood glucose, adherence to recommended health promoting behaviours, quality of life, satisfaction, pregnancy-induced hypertension, cues to action, perceived benefits, perceived barriers, perceived severity, perceived susceptibility, and satisfactory knowledge. However, there was a trend towards the self-management programmes being similar to usual care in improving age of neonate at delivery, premature rupture of membranes, postpartum haemorrhage, and days of maternal and neonatal hospitalisation. The impact on the rates of caesarean delivery is conflicting.

These findings align with the reviews of self-management programmes for other diabetic populations including adults, adolescents and/or children with type 1 or type 2 diabetes mellitus. Self-management programmes were efficacious and cost effective for facilitation of self-management, improvements in patients' knowledge, skills, and motivation, and improved biomedical, behavioural, and psychosocial outcomes [55]. Self-management programmes had a greater impact on glycaemic outcomes than on mental health outcomes which were rarely assessed [56]. Blood glucose control, diabetes knowledge, body weight, blood pressure, low density lipoprotein cholesterol, mean arterial pressure, anxiety and depression, diabetes distress, sedentary behaviours, quality of life, self-efficacy, self-care, self-management skills, and treatment satisfaction were improved following diabetes self-management programmes [57]. Self-management produced better improvements in glycated haemoglobin, diabetes knowledge, self-efficacy, self-management behaviours, depression, quality of life and patient satisfaction compared with routine care [58]. Self-management programmes were better than routine care for fasting blood glucose, blood pressure, body mass index, self-efficacy, diabetes knowledge; but were similar for self-management practices, physical activity, diabetes distress, and depression [59].

The conflicting results of the impact of self-management programmes for some outcomes in our review could be due to the different content, intensity, and delivery of the self-management programmes in few studies. Other reviews of diabetes self-management programmes have made similar observations and reported very few and differing studies of diverse diabetes self-management programmes which make interpretation and generalisation of findings difficult [57, 59]. The recommendation that diabetes self-management programmes be tailored acknowledging individual and cultural needs [60] might increase effectiveness and consistency of findings. Determining and utilising active components and core outcomes of diabetes self-management programmes could enhance effectiveness and consistency of findings. Self-management programmes focus on lifestyle changes, health behaviour change, and daily self-management of conditions [61–64] which could explain the consistent superior effectiveness of the community-based self-management programmes on health behaviour related outcomes when compared with routine care in our review.

Our results show that community-based medical nutrition/diet therapy interventions were more effective than routine care in improving postprandial blood glucose levels; which can be a proxy for good adherence to nutritional therapy [65]. The effectiveness of the interventions was less certain for the other outcomes because only one study reported each of them. Overall, there was a trend towards community-based medical nutrition/diet therapy interventions being superior to or as effective as usual care in the other maternal outcomes and all neonatal outcomes. Intensive nutritional interventions tended to be more effective than usual care in the greatest number of outcomes. Although group nutritional counselling was as effective as individual nutritional counselling in improving diabetes knowledge, group counselling had an additional advantage of saving dietitian hours.

Overall, our review found no evidence that any specific community-based nutritional therapy was superior to the other. This could be because any diet based on low glycaemic index diet, high in complex carbohydrate and fibre, low in simple sugar and low in saturated fat produce positive outcomes in GDM including reduction in blood glucose, prevention of insulin resistance, and attenuating excess foetal fat accretion [65–70]. Low glycaemic index diet is known to reduce two hour post prandial glucose, fasting blood glucose, and lipid profile in patients with GDM [71]. This aligns with our review which found that glycaemic control was similar between low glycaemic index and conventional high fibre diets. Low carbohydrate diet is not better than high complex carbohydrate diet [72]. Low carbohydrate diet can stimulate higher fat intake exacerbating maternal insulin resistance due to increased free fatty acids [70, 73, 74]. High fibre and low-fat intake increase gut microbiota diversity and richness which reduce insulin resistance and inflammatory response [75]. Caloric restriction is generally advised only for overweight and obese pregnant women with GDM [65, 66]. However, there is no consensus on specific diet characteristics including carbohydrate distribution and quantity due to the limited number of high quality clinical trials and the complexity that such interventions and studies will warrant [65, 66, 70, 76].

The strength of evidence for the effectiveness of community-based exercise/physical activity was limited due to the paucity of studies (less than two studies for each outcome). There was a trend for physical activity/exercise interventions being superior in improving postprandial blood glucose levels than routine care and no treatment. Other outcomes were less consistent but overall physical activity/exercise interventions tended to be better than or as effective as routine care in improving fasting blood glucose, glycaemic control, mean glucose levels, proportion requiring insulin, quantity of insulin required, time to insulin use, new-born birthweight, rates of caesarean section, rates of preterm birth, maternal body mass index, rates of low birth weight, macrosomia, rates of pregnancy and birth complications, need for pharmacological therapy, maternal body mass and body fat percentage, neonatal Apgar scores, ponderal index. Neonatal body mass index was the only outcome reported to be better with routine obstetric care in one study. There is limited direct evidence with which to compare these findings. A systematic review which shows that physical activity during pregnancy improves fasting and postprandial glucose, as well as glycated haemoglobin in pregnant women with GDM, had four of the included six studies combining exercise with diet [77].

Most reviews have focused on the prevention of GDM using exercise/physical activity. Although previous reviews found insufficient evidence that exercise/physical activity during pregnancy is effective in reducing the development of GDM [78, 79], more recent systematic reviews have found that exercise during pregnancy reduces the risk of developing GDM [80–82]. The greatest benefit of physical activity in reducing the risk of GDM occurs before pregnancy [83]. Findings from our review and previous reviews have not shown any evidence for the superiority of any exercise type. However, guidelines recommend both aerobic and resistance exercise at a moderate intensity, a minimum of three times per week for 30-60 minutes each time [84]. The exercise/physical activity interventions in our review were aerobic, resistance, or combined aerobic and resistance exercise which may align with this recommendation. Exercise/physical activity provides an alternative pathway of glucose uptake to insulin activated transport via muscular contraction that ultimately stimulates glucose transport; directly increases the biogenesis of GLUT4 which is an insulin-regulated glucose transporter that is responsible for insulin-regulated glucose uptake into fat and muscle cells; and can strengthen and compensate for defects in insulin signalling; which improve glycaemic control [85].

Our review found very limited evidence that community-based diet and exercise was better than diet alone or routine obstetric care in improving maternal and neonatal outcomes particularly maternal and neonatal weight. Evidence suggest that dietary improvements and physical activity are effective in managing hyperglycaemia and the associated sequelae [86].

Similarly, our review found very limited evidence that combined calcium and vitamin D supplement therapy was better than placebo in reducing the rates of caesarean delivery, maternal hospitalisation, hyperbilirubinaemia, newborn hospitalisation, and preventing macrosomia. Evidence suggests that multivitamins containing vitamin D reduce the risk of preeclampsia which was not found with administering only vitamins C and E [66, 87]. There is some evidence that vitamin D supplements improve insulin sensitivity in women with GDM, and an intake of 900-1000mg of calcium per day is recommended for all pregnant women [66, 87]. Calcium is considered important because the hyperglycaemia resulting from GDM is associated with neonatal hypocalcaemia, hypoglycaemia, hyperbilirubinemia, polycythaemia and respiratory distress syndrome [88].

We found very limited evidence that continuous glucose monitoring added to routine obstetric care reduced risk of pre-eclampsia and caesarean delivery, and reduced birth weight and neonatal complications better than routine obstetric care only. A recent meta-analysis of six RCTs found that continuous glucose monitoring was associated with lower glycated haemoglobin, less gestational weight gain, lower birth weight compared to standard blood glucose monitoring. However, there were no differences between continuous glucose monitoring and standard blood glucose monitoring for gestational age newborns, gestational age at birth, preterm deliveries, shoulder dystocia, neonatal hypoglycaemia, Apgar at five minutes, admission to neonatal intensive care unit, neonatal jaundice, and neonatal mortality [89].

### Strengths and limitations

To the authors’ knowledge, this systematic review is the first to investigate community-based non-pharmacological interventions for pregnant women with GDM. Moreover, the concept of community-based interventions is evolving and can be diverse since ‘community’ can be conceptualised as the setting, target, agent, or the resource [90]. The inclusion of all relevant primary studies on GDM regardless of study design and publication date; plus, the grading of the strength of each outcome strengthens the evidence-base on which the findings of this review are based.

This review is limited by the lack of a consensus definition and diagnostic criteria for GDM in individual studies. This review used the most widely accepted definition of GDM as hyperglycaemia during pregnancy. This implies all cases of hyperglycaemia during pregnancy including true GDM (which develops later during pregnancy) and previously undetected pre-existing diabetes (which is also referred to as ‘overt diabetes’ and is often identified in early pregnancy, and comprises both pre-gestational type 2 diabetes mellitus and type 1 diabetes mellitus) [11]. Therefore, the findings from this systematic review may not be exclusive to GDM. This may not be a limitation per se since preventive strategies for type 2 diabetes may also be successful in the prevention of GDM [11]. The inclusion of observational studies alongside RCTs is a limitation which was addressed by the grading of the overall quality of evidence for each outcome. The heterogeneity in intervention content, study design and settings prohibit the endorsement of any specific components of the community-based non-pharmacological interventions.

## Conclusions

Community-based non-pharmacological interventions were more effective or as effective as routine obstetric care in improving the most maternal and neonatal outcomes; and could be delivered by a broad range of health professionals. Of the six interventions identified, self-management programmes and medical nutrition/diet therapy had the strongest evidence for the most promising outcomes – on postprandial blood glucose levels and health behaviour related outcomes. There is need for more research on the effectiveness of these interventions whilst focusing on core GDM outcomes [91–94] and the active components of these interventions. There may be need to compare the clinical and cost-effectiveness of these interventions with hospital-based interventions.

## Supplementary Information

**Additional file 1.**

## Data Availability

The review protocol is freely available on PROSPERO. Further information can be obtained via request to the corresponding author.

## Abbreviations

GDM: Gestational Diabetes Mellitus
PRISMA: Preferred Reporting Items for Systematic reviews and Meta-Analyses
SWiM: Synthesis without meta-analysis in systematic reviews
MeSH: Medical Subject Heading
RCT: Randomised Controlled Trial
CIT: Cell phone–Internet Technology
LGI: Low–Glycaemic Index
DASH: Dietary Approaches to Stop Hypertension
MNT: Medical Nutrition Therapy

## References

[1] Craig L, Sims R, Glasziou P, Thomas R (2020). Women’s experiences of a diagnosis of gestational diabetes mellitus: a systematic review. BMC Pregnancy Childbirth..

[2] McIntyre HD, Catalano P, Zhang C, Desoye G, Mathiesen ER, Damm P (2019). Gestational diabetes mellitus. Nat Rev Dis Prim..

[3] Damm P, Houshmand-Oeregaard A, Kelstrup L, Lauenborg J, Mathiesen ER, Clausen TD (2016). Gestational diabetes mellitus and long-term consequences for mother and offspring: a view from Denmark. Diabetologia..

[4] Hayashi A, Oguchi H, Kozawa Y, Ban Y, Shinoda J, Suganuma N (2018). Daily walking is effective for the management of pregnant women with gestational diabetes mellitus. J Obstet Gynaecol Res..

[5] Dirar AM, Doupis J (2017). Gestational diabetes from A to Z. World J Diabetes..

[6] Zhu Y, Zhang C (2016). Prevalence of gestational diabetes and risk of progression to type 2 diabetes: a global perspective. Curr Diab Rep..

[7] Hall D, Du Toit M, Mason D, Conradie M (2015). Diabetes mellitus in pregnancy, still changing. J Endocrinol Metab Diabetes South Africa..

[8] Grzelak T, Janicka E, Kramkowska M, Walczak M, Czyżewska K (2013). Cukrzyca ciążowa–skutki niewyrównania i podstawy regulacji glikemii. Now Lek..

[9] Di Guardo F, Currò JM, Valenti G, Rossetti P, Di Gregorio LM, Conway F, et al. Non-pharmacological management of gestational diabetes: The role of myo-inositol. J Complement Integr Med. 2020;17(2). 10.1515/jcim-2019-0111. 10.1515/jcim-2019-0111 31527297

[10] Guo X, Shu J, Fu X, Chen X, Zhang L, Ji M (2019). Improving the effectiveness of lifestyle interventions for gestational diabetes prevention: a meta-analysis and meta-regression. BJOG An Int J Obstet Gynaecol..

[11] Zito G, Della Corte L, Giampaolino P, Terzic M, Terzic S, Di Guardo F (2020). Gestational diabetes mellitus: Prevention, diagnosis and treatment. A fresh look to a busy corner. J Neonatal Perinatal Med..

[12] Gilbert L, Gross J, Lanzi S, Quansah DY, Puder J, Horsch A (2019). How diet, physical activity and psychosocial well-being interact in women with gestational diabetes mellitus: an integrative review. BMC Pregnancy Childbirth..

[13] Moisés ECD (2013). Multidisciplinary Care of Pregnant Women with Gestational Diabetes Mellitus: Non-Pharmacological Strategies to Improve Maternal and Perinatal Outcomes. Gestation Diabetes-Causes, Diagnosis Treat.

[14] Mariani HS, Layden BT, Aleppo G (2017). Continuous glucose monitoring: a perspective on its past, present, and future applications for diabetes management. Clin Diabetes..

[15] Mirfeizi M, Mehdizadeh Tourzani Z, Asghari Jafarabadi M, Moghimi Hanjani S, Hasanzad M (2017). Health education in gestational diabetes mellitus and quality of life. J Midwifery Reprod Heal..

[16] Adesina N, Dogan H, Green S, Tsofliou F (2021). Effectiveness and Usability of Digital Tools to Support Dietary Self-Management of Gestational Diabetes Mellitus: A Systematic Review. Nutrients..

[17] Karavasileiadou S, Almegewly W, Alanazi A, Alyami H, Chatzimichailidou S (2022). Self-management and self-efficacy of women with gestational diabetes mellitus: a systematic review. Glob Health Action..

[18] Carolan-Olah MC (2016). Educational and intervention programmes for gestational diabetes mellitus (GDM) management: An integrative review. Collegian..

[19] Nielsen KK, de Courten M, Kapur A (2012). Health system and societal barriers for gestational diabetes mellitus (GDM) services-lessons from World Diabetes Foundation supported GDM projects. BMC Int Health Hum Rights..

[20] Kolu P, Raitanen J, Rissanen P, Luoto R (2012). Health care costs associated with gestational diabetes mellitus among high-risk women–results from a randomised trial. BMC Pregnancy Childbirth..

[21] Murphy JW (2014). Community-based interventions: Philosophy and action.

[22] Igwesi-Chidobe CN, Emmanuel GN, Okezue OC (2021). Community-based non-pharmacological interventions for improving pain, disability and quality of life in pregnant women with musculoskeletal conditions: protocol for a systematic review with meta-analyses. BMJ Open..

[23] Page MJ, Moher D, Bossuyt PM, Boutron I, Hoffmann TC, Mulrow CD (2021). PRISMA 2020 explanation and elaboration: updated guidance and exemplars for reporting systematic reviews. BMJ..

[24] Campbell M, McKenzie JE, Sowden A, Katikireddi SV, Brennan SE, Ellis S (2020). Synthesis without meta-analysis (SWiM) in systematic reviews: reporting guideline. BMJ..

[25] Higgins JPT, Thomas J, Chandler J, Cumpston M, Li T, Page MJ (2019). Cochrane handbook for systematic reviews of interventions.

[26] Page MJ, McKenzie JE, Bossuyt PM, Boutron I, Hoffmann TC, Mulrow CD (2021). The PRISMA 2020 statement: an updated guideline for reporting systematic reviews. BMJ..

[27] Cochrane Consumers and Communication Review Group (2016). data extraction template for Cochrane reviews.

[28] Higgins JPT, Altman DG, Gøtzsche PC, Jüni P, Moher D, Oxman AD (2011). The Cochrane Collaboration’s tool for assessing risk of bias in randomised trials. BMJ..

[29] Daniel JA, Dikki CE (2014). Aerobic dance exercise improves blood glucose level in pregnant women with gestational diabetes mellitus science. African J Phys Heal Educ Recreat Danc..

[30] de Barros MC, Lopes MAB, Francisco RPV, Sapienza AD, Zugaib M (2010). Resistance exercise and glycemic control in women with gestational diabetes mellitus. Am J Obstet Gynecol..

[31] Kokic IS, Ivanisevic M, Biolo G, Simunic B, Kokic T, Pisot R (2018). Combination of a structured aerobic and resistance exercise improves glycaemic control in pregnant women diagnosed with gestational diabetes mellitus. A randomised controlled trial. Women Birth..

[32] Mohebbi B, Tol A, Sadeghi R, Mohtarami SF, Shamshiri A (2019). Self-management Intervention Program Based on the Health Belief Model (HBM) among Women with Gestational Diabetes Mellitus: A Quazi-Experimental Study. Arch Iran Med..

[33] Yang P, Lo W, He Z, Xiao X (2018). Medical nutrition treatment of women with gestational diabetes mellitus by a telemedicine system based on smartphones. J Obstet Gynaecol Res..

[34] El Sayed Ibrahim R, Mousa Saber N (2019). Impact of self-care program for gestational diabetic women on pregnancy outcomes. Am J Nurs Res..

[35] Bartholomew ML, Soules K, Church K, Shaha S, Burlingame J, Graham G (2015). Managing diabetes in pregnancy using cell phone/internet technology. Clin Diabetes..

[36] Yu F, Lv L, Liang Z, Wang Y, Wen J, Lin X (2014). Continuous glucose monitoring effects on maternal glycemic control and pregnancy outcomes in patients with gestational diabetes mellitus: a prospective cohort study. J Clin Endocrinol Metab..

[37] Sunsaneevithayakul P, Ruangvutilert P, Sutanthavibul A, Kanokpongsakdi S, Boriboohirunsarn D, Raengpetch Y (2004). Effect of 3-day intensive dietary therapy during admission in women after diagnosis of gestational diabetes mellitus. J Med Assoc Thail..

[38] Hu Z-G, Tan R-S, Jin D, Li W, Zhou X-Y (2014). A low glycemic index staple diet reduces postprandial glucose values in Asian women with gestational diabetes mellitus. J Investig Med..

[39] Louie JCY, Markovic TP, Perera N, Foote D, Petocz P, Ross GP (2011). A randomized controlled trial investigating the effects of a low–glycemic index diet on pregnancy outcomes in gestational diabetes mellitus. Diabetes Care..

[40] Asemi Z, Samimi M, Tabassi Z, Esmaillzadeh A (2014). The effect of DASH diet on pregnancy outcomes in gestational diabetes: a randomized controlled clinical trial. Eur J Clin Nutr..

[41] Grant SM, Wolever TMS, O’Connor DL, Nisenbaum R, Josse RG (2011). Effect of a low glycaemic index diet on blood glucose in women with gestational hyperglycaemia. Diabetes Res Clin Pract..

[42] Artal R, Catanzaro RB, Gavard JA, Mostello DJ, Friganza JC (2007). A lifestyle intervention of weight-gain restriction: diet and exercise in obese women with gestational diabetes mellitus. Appl Physiol Nutr Metab..

[43] Kgosidialwa O, Egan AM, Carmody L, Kirwan B, Gunning P, Dunne FP (2015). Treatment with diet and exercise for women with gestational diabetes mellitus diagnosed using IADPSG criteria. J Clin Endocrinol Metab..

[44] Wang C, Zhu W, Wei Y, Feng H, Su R, Yang H (2015). Exercise intervention during pregnancy can be used to manage weight gain and improve pregnancy outcomes in women with gestational diabetes mellitus. BMC Pregnancy Childbirth..

[45] Mendelson SG, Mcneese-Smith D, Koniak-Griffin D, Nyamathi A, Lu MC (2008). A community-based parish nurse intervention program for Mexican American Women with gestational diabetes. JOGNN J Obstet Gynecol Neonatal Nurs..

[46] Ural A, Kizilkaya Beji N (2021). The effect of health-promoting lifestyle education program provided to women with gestational diabetes mellitus on maternal and neonatal health: a randomized controlled trial. Psychol Health Med..

[47] Kolivand M, Rahimi MA, Keramat A, Shariati M, Emamian MH (2019). Effect of a new self-care guide package on maternal and neonatal outcomes in gestational diabetes: A randomized control trial. J Diabetes..

[48] Al-Hashmi I, Hodge F, Nandy K, Thomas E, Brecht M-L (2018). The effect of a self-efficacy-enhancing intervention on perceived self-efficacy and actual adherence to healthy behaviours among women with gestational diabetes mellitus. Sultan Qaboos Univ Med J..

[49] Shi M, Liu Z-L, Steinmann P, Chen J, Chen C, Ma X-T (2016). Medical nutrition therapy for pregnant women with gestational diabetes mellitus—a retrospective cohort study. Taiwan J Obstet Gynecol..

[50] Perichart-Perera O, Balas-Nakash M, Parra-Covarrubias A, Rodriguez-Cano A, Ramirez-Torres A, Ortega-González C (2009). A medical nutrition therapy program improves perinatal outcomes in Mexican pregnant women with gestational diabetes and type 2 diabetes mellitus. Diabetes Educ..

[51] Murphy A, Guilar A, Donat D (2004). Nutrition education for women with newly diagnosed gestational diabetes mellitus: small-group vs. individual counselling. Can J diabetes..

[52] Karamali M, Asemi Z, Ahmadi-Dastjerdi M, Esmaillzadeh A (2016). Calcium plus vitamin D supplementation affects pregnancy outcomes in gestational diabetes: randomized, double-blind, placebo-controlled trial. Public Health Nutr..

[53] Tian Y, Zhang S, Huang F, Ma L (2021). Comparing the Efficacies of Telemedicine and Standard Prenatal Care on Blood Glucose Control in Women With Gestational Diabetes Mellitus: Randomized Controlled Trial. JMIR mHealth uHealth..

[54] Barati Z, Iravani M, Karandish M, Haghighizadeh MH, Masihi S (2021). The effect of oat bran consumption on gestational diabetes: A randomized controlled clinical trial. BMC Endocr Disord..

[55] Chatterjee S, Davies MJ, Heller S, Speight J, Snoek FJ, Khunti K (2018). Diabetes structured self-management education programmes: a narrative review and current innovations. Lancet Diabetes Endocrinol..

[56] Hermanns N, Ehrmann D, Finke-Groene K, Kulzer B (2020). Trends in diabetes self-management education: where are we coming from and where are we going? A narrative review. Diabet Med..

[57] Vas A, Devi ES, Vidyasagar S, Acharya R, Rau NR, George A (2017). Effectiveness of self-management programmes in diabetes management: A systematic review. Int J Nurs Pract..

[58] Amagyei A, Meal A, Shaw I, Adams GG. Effectiveness of Community Health Worker-led Diabetes Self-Management Education on Type 2 diabetes patients: A Systematic Review and Meta-Analysis. Int J Diabetes. 2020;1(2):40-50.

[59] Werfalli M, Raubenheimer PJ, Engel M, Musekiwa A, Bobrow K, Peer N (2020). The effectiveness of peer and community health worker-led self-management support programs for improving diabetes health-related outcomes in adults in low-and-middle-income countries: a systematic review. Syst Rev..

[60] Haas L, Maryniuk M, Beck J, Cox CE, Duker P, Edwards L (2014). National standards for diabetes self-management education and support. Diabetes Care..

[61] Barlow J, Wright C, Sheasby J, Turner A, Hainsworth J (2002). Self-management approaches for people with chronic conditions: a review. Patient Educ Couns..

[62] Grady PA, Gough LL (2014). Self-management: a comprehensive approach to management of chronic conditions. Am J Public Health..

[63] Allegrante JP, Wells MT, Peterson JC (2019). Interventions to support behavioral self-management of chronic diseases. Annu Rev Public Health..

[64] Newman S, Steed L, Mulligan K (2004). Self-management interventions for chronic illness. Lancet..

[65] Moreno-Castilla C, Mauricio D, Hernandez M (2016). Role of medical nutrition therapy in the management of gestational diabetes mellitus. Curr Diab Rep..

[66] Vasile FC, Preda A, Ștefan AG, Vladu MI, Forțofoiu M-C, Clenciu D (2021). An Update of Medical Nutrition Therapy in Gestational Diabetes Mellitus. J Diabetes Res..

[67] Mahajan A, Donovan LE, Vallee R, Yamamoto JM (2019). Evidenced-based nutrition for gestational diabetes mellitus. Curr Diab Rep..

[68] Wang H-K, Cheng D-C, Yang Y-M, Wang X-H, Chen Y, Zhang L (2021). The Role of High-Content Complex Dietary Fiber in Medical Nutrition Therapy for Gestational Diabetes Mellitus. Front Pharmacol..

[69] Tsirou E, Grammatikopoulou MG, Theodoridis X, Gkiouras K, Petalidou A, Taousani E (2019). Guidelines for medical nutrition therapy in gestational diabetes mellitus: systematic review and critical appraisal. J Acad Nutr Diet..

[70] Hernandez TL, Anderson MA, Chartier-Logan C, Friedman JE, Barbour LA (2013). Strategies in the nutritional management of gestational diabetes. Clin Obstet Gynecol..

[71] Filardi T, Panimolle F, Crescioli C, Lenzi A, Morano S (2019). Gestational diabetes mellitus: The impact of carbohydrate quality in diet. Nutrients..

[72] Moreno-Castilla C, Hernandez M, Bergua M, Alvarez MC, Arce MA, Rodriguez K (2013). Low-carbohydrate diet for the treatment of gestational diabetes mellitus: a randomized controlled trial. Diabetes Care..

[73] Hernandez TL, Brand-Miller JC (2018). Nutrition therapy in gestational diabetes mellitus: time to move forward. Diabetes Care..

[74] Hernandez TL, Mande A, Barbour LA (2018). Nutrition therapy within and beyond gestational diabetes. Diabetes Res Clin Pract..

[75] Ponzo V, Fedele D, Goitre I, Leone F, Lezo A, Monzeglio C (2019). Diet-gut microbiota interactions and gestational diabetes mellitus (GDM). Nutrients..

[76] Meloncelli N, Wilkinson SA, de Jersey S (2020). Searching for utopia, the challenge of standardized medical nutrition therapy prescription in gestational diabetes mellitus management: a critical review. Seminars in reproductive medicine.

[77] Laredo-Aguilera JA, Gallardo-Bravo M, Rabanales-Sotos JA, Cobo-Cuenca AI, Carmona-Torres JM (2020). Physical activity programs during pregnancy are effective for the control of gestational diabetes mellitus. Int J Environ Res Public Health..

[78] Yin Y, Li X, Tao T, Luo B, Liao S (2014). Physical activity during pregnancy and the risk of gestational diabetes mellitus: a systematic review and meta-analysis of randomised controlled trials. Br J Sports Med..

[79] Ruchat S, Mottola MF (2013). The important role of physical activity in the prevention and management of gestational diabetes mellitus. Diabetes Metab Res Rev..

[80] Sanabria-Martínez G, García-Hermoso A, Poyatos-León R, Álvarez-Bueno C, Sánchez-López M, Martínez-Vizcaíno V (2015). Effectiveness of physical activity interventions on preventing gestational diabetes mellitus and excessive maternal weight gain: a meta-analysis. BJOG An Int J Obstet Gynaecol..

[81] Russo LM, Nobles C, Ertel KA, Chasan-Taber L, Whitcomb BW (2015). Physical activity interventions in pregnancy and risk of gestational diabetes mellitus: a systematic review and meta-analysis. Obstet Gynecol..

[82] Suhail ARD, Furuya-Kanamori L, Toft E, Musa OAH, Mohamed AM, Clark J (2020). Physical activity in pregnancy prevents gestational diabetes: A meta-analysis.

[83] Tobias DK, Zhang C, Van Dam RM, Bowers K, Hu FB (2011). Physical activity before and during pregnancy and risk of gestational diabetes mellitus: a meta-analysis. Diabetes Care..

[84] Padayachee C, Coombes JS (2015). Exercise guidelines for gestational diabetes mellitus. World J Diabetes..

[85] Wang C, Guelfi KJ, Yang H-X (2016). Exercise and its role in gestational diabetes mellitus. Chronic Dis Transl Med..

[86] Bain E, Crane M, Tieu J, Han S, Crowther CA, Middleton P. Diet and exercise interventions for preventing gestational diabetes mellitus. Cochrane database Syst Rev. 2015;4(CD010443). 10.1002/14651858.CD010443.pub2. 10.1002/14651858.CD010443.pub2 25864059

[87] Dolatkhah N, Hajifaraji M, Shakouri SK (2018). Nutrition therapy in managing pregnant women with gestational diabetes mellitus: a literature review. J Fam Reprod Heal..

[88] Carreiro MP, Nogueira AI, Ribeiro-Oliveira A (2018). Controversies and advances in gestational diabetes—an update in the era of continuous glucose monitoring. J Clin Med..

[89] García-Moreno RM, Benítez-Valderrama P, Barquiel B, González Pérez-de-Villar N, Hillman N, Lora Pablos D (2022). Efficacy of continuous glucose monitoring on maternal and neonatal outcomes in gestational diabetes mellitus: a systematic review and meta-analysis of randomized clinical trials. Diabet Med..

[90] McLeroy KR, Norton BL, Kegler MC, Burdine JN, Sumaya CV (2003). Community-based interventions. Am J Public Health..

[91] Egan AM, Bogdanet D, Griffin TP, Kgosidialwa O, Cervar-Zivkovic M, Dempsey E (2020). A core outcome set for studies of gestational diabetes mellitus prevention and treatment. Diabetologia..

[92] Bashir M, Syed A, Furuya-Kanamori L, Musa OAH, Mohamed AM, Skarulis M (2021). Core outcomes in gestational diabetes for treatment trials: the Gestational Metabolic Group treatment set. Obes Sci Pract..

[93] Bain E, Middleton P, Crowther CA (2016). Progressing towards standard outcomes in gestational diabetes Cochrane reviews and randomised trials. Aust New Zeal J Obstet Gynaecol..

[94] O’Reilly SL, Leonard Y, Dasgupta K, Terkildsen Maindal H (2020). Diabetes after pregnancy prevention trials: systematic review for core outcome set development. Matern Child Nutr..

