# Supplementary material for: Community-based non-pharmacological interventions for pregnant women with gestational diabetes mellitus: a systematic review
Source: BMC Womens Health. 2022 Nov 29;22:482. doi: 10.1186/s12905-022-02038-9 (PMC9710028; doi:10.1186/s12905-022-02038-9)
Supplement: Supplementary file 1 — Additional file 1. [file 12905_2022_2038_MOESM1_ESM.docx]

## **APPENDIX**

SEARCH STRATEGIES

PUBMED

1. ((Pregnancy) OR (Pregnancies) OR (Gestation) OR (Pregnant Women) OR (Pregnant Woman) OR (Woman, Pregnant) OR (Women, Pregnant))
2. (birth OR prenatal OR perinatal OR parturition) OR tiab (“delivery, obstetric” OR “cesarean section” OR “extraction, obstetrical”) OR tiab (“labor, induced” OR antenatal OR puerperium OR postnatal)
3. (pregnancy or pregnant or gestational or intrauterine or birth)
4. 1 OR 2 OR 3
5. (Gestational diabetes mellitus OR GDM OR gestational diabetes OR pregnancy-induced diabetes mellitus OR gestational OR pregnancy glucose intolerance OR pregnancy hyperglycaemia OR pregnancy glycaemic index OR Pregnancy-Induced Diabetes OR Diabetes Gestational)
6. “gestational diabetes” OR “gestational diabetic” OR “diabetes in pregnancy” OR pregnancy diabetes mellitus
7. Diabetes Gestational OR gestational diabetes OR tiab (pregnancy-induced diabetes) OR tiab pregnancy-induced diabetes mellitus tiab OR GDM.
8. 5 OR 6 OR 7
9. Community Based Participatory Research* or Community Based Participatory Research study* or Community Trial*
10. “Lay health worker” OR “community based” OR “Health workers” OR “Health worker” OR “aides”
11. “Community based treatment” OR “community-directed” OR “community engagement” OR “public health program”
12. 9 OR 10 OR 11
13. (non-pharmacologic*[tw] OR nonpharmacologic*[tw] OR "Art Therapy"[mesh] OR "art therapy"[tw] OR "Music Therapy"[mesh] OR "music therapy"[tw] OR "Music"[mesh] OR "music"[tw] OR "Art"[mesh] OR "Singing"[Mesh] OR "singing"[tw] OR "singing therapy"[tw] OR "Exercise"[mesh] OR "exercise"[tw] OR "exercises"[tw] OR "walking"[tw] OR "walk"[tw] OR "Stair Climbing"[tw] OR outdoor*[tw] OR "Animal Assisted Therapy"[Mesh] OR "Animal Assisted Therapy"[tw] OR "Animal Assisted Therapies"[tw] OR "Animal Facilitated Therapy"[tw] OR "Pet Therapy"[tw] OR "Pet Therapies"[tw] OR "Pet Facilitated Therapy"[tw] OR "Sensory Art Therapies"[mesh] OR "Sensory Art Therapies"[tw] OR "sensory stimulation"[tw] OR sensory stimulat*[tw] OR "Smell"[tw] OR "smell"[tw] OR "Sensory integration"[tw] OR "Sensory Thresholds"[Mesh] OR "Sensation/physiology"[Mesh] OR "Aromatherapy"[mesh] OR aromatherap*[tw] OR "Recreation Therapy"[mesh] OR "recreation therapy"[tw] OR "recreational therapy"[tw] OR "Behavior Therapy"[Mesh] OR "Behavior Therapy"[tw] OR "Behavioral therapy"[tw] OR "Behaviour Therapy"[tw] OR "Behavioural therapy"[tw] OR "Anger Management Therapy"[tw] OR "Applied Behavior Analysis"[tw] OR "Applied Behaviour Analysis"[tw] OR "Aversive Therapy"[tw] OR "Psychology Biofeedback"[tw] OR "Sensory Feedback"[tw] OR "Neurofeedback"[tw] OR "Cognitive Remediation"[tw] OR "Cognitive Therapy"[tw] OR "Acceptance and Commitment Therapy"[tw] OR "Mindfulness"[tw] OR "Psychologic Desensitization"[tw] OR "Psychological Desensitization"[tw] OR "Eye Movement Desensitization Reprocessing"[tw] OR "EMDR"[tw] OR "Implosive Therapy"[tw] OR "Virtual Reality Exposure Therapy"[tw] OR "Relaxation Therapy"[tw] OR "Meditation"[tw] OR "Sleep Phase Chronotherapy"[tw] OR "Reminiscing therapy"[tw] OR reminisc*[tw] OR "pleasant activities"[tw] OR "pleasant activity"[tw] OR "Photos"[tw] OR "Photographs"[tw] OR "Photo"[tw] OR "Photograph"[tw] OR "Narration"[tw] OR "story telling"[tw] OR "telling stories"[tw] OR "narration"[tw] OR "watching football"[tw] OR "Color Therapy"[mesh] OR "color therapy"[tw] OR "magic table"[tw] OR ("magic"[tw] AND table*[tw]) OR fiddle*[tw] OR "Play and Playthings"[Mesh] OR Bingo*[tw] OR "Recreation"[mesh] OR "Dance Therapy"[mesh] OR "dance therapy"[tw] OR "Play Therapy"[mesh] OR "play therapy"[tw])
14. Psychotherapy OR Cognitive Therapy OR Behavior Therapy OR Aromatherapy OR Massage OR Music Therapy OR Animal Assisted Therapy OR Exercise OR Art Therapy OR Horticultural Therapy OR Occupational Therapy OR Telerehabilitation OR Therapy OR Computer-Assisted OR; Dance Therapy OR Play Therapy OR Reality Therapy OR Recreation Therapy OR non pharmacological OR non drug OR light therap* OR Snoezelen OR multimodality therap* OR multisensory OR doll therapy OR robot therapy OR cognitive training
15. rehabilitat* or physiotherapy* or physical therapy* or occupation* or acupuncture or social work or orthotics* or cognitive therapy* or behavior therapy* or counseling* or nutrition* or diet* or food or physical activity or glycemic control monitoring or self-monitoring of blood glucose or health education programmes
16. 13 OR 14 OR 15
17. 4 AND 8 AND 12 AND 16

CINAHL

1. (MH "Pregnancy+") OR (MM "Pregnancies+") OR (MM "Gestation+") OR (MM "Pregnant Women+") OR (MM "Pregnant Woman+") OR (MM "Woman, Pregnant+") OR (MM "Women, Pregnant+")
2. (MH "Childbirth+") OR (MM "prenatal+") OR (MM "perinatal+") OR (MM "parturition+") OR (MM "delivery, obstetric+") OR (MM "cesarean section+") OR (MM "labor, induced+") OR (MM "antenatal+") OR (MM "puerperium+") OR (MM "postnatal+") OR (MH "Pregnancy Outcomes") OR (MH "Pregnancy, High Risk+")
3. S1 OR S2
4. (MH "Diabetes Mellitus, Gestational") OR (MM "Pregnancy in Diabetes+") OR "gestational, blood glucose metabolism disorder" OR (MM "Hyperglycemia+")
5. (MM "Glucose Intolerance") OR (MH "Pregnancy in Diabetes+") OR "Pregnancy hyperglycaemia" OR "Gestational diabetes mellitus" OR "GDM" OR "gestational diabetes" OR "pregnancy-induced diabetes mellitus" OR "pregnancy glucose intolerance" OR "pregnancy glycaemic index" OR "Diabetes in pregnancy" OR "Diabetes Gestational" OR "pregnancy diabetes mellitus"
6. S4 OR S5
7. (MM "Rehabilitation, Community-Based") OR "Community based intervention" OR (MM "Community Role") OR (MM "Environment and Public Health+") OR (MM "Community Service") OR (MM "Community Programs")
8. (MM "Community Health Workers") OR "Community Based Participatory Research" OR "Community Based Participatory Research study" OR "Community Trial" OR "Lay health worker" OR "Health workers" OR "Health worker" OR "aides" OR "Community based treatment" OR "community-directed" OR "community engagement" OR "public health program"
9. S7 OR S8
10. "non-pharmacological interventions" OR (MH "Manual Therapy+") OR (MH "Diet Therapy+") OR (MM "Psychotherapy+") OR (MM "Cognitive Therapy+") OR (MH "Rehabilitation+") OR (MH "Self Care+") OR (MH "Physical Therapy+") OR (MH "Physical Activity") OR (MH "Glycemic Control") OR "glycemic control monitoring" OR (MH "Blood Glucose Self-Monitoring") OR (MM "Blood Glucose Monitoring+") OR "self monitoring of blood glucose" OR "self-monitoring of blood glucose" OR blood glucose self monitoring OR blood glucose self-monitoring OR (MM "Health Education+") OR "health education programmes" OR "Physiotherapy" OR "occupation" OR "acupuncture" OR "social work" OR "orthotics" OR "counseling" OR "nutrition" OR "diet" OR "food" OR "non-drug" OR "light therapy" OR "Snoezelen" OR "multimodality therapy"
11. (MM "Behavior Therapy+") OR (MM "Cognitive Therapy+") OR (MM "Exercise Promotion (Iowa NIC)") OR (MM "Acceptance and Commitment Therapy") OR (MM "Recreational Therapy") OR "Exercise" OR "exercises" OR "walking" OR "walk" OR "Stair Climbing" OR "outdoor" OR "Sensory Art Therapies" OR "Sensory Art Therapies" OR "sensory stimulation" OR "Sensory integration" OR "Sensory Thresholds" OR "Sensation" OR "physiology" OR "Aromatherapy" OR "Recreation Therapy" OR "Behavior Therapy" OR "Behavioral therapy" OR "Aversive Therapy" OR "Psychology Biofeedback" OR "Sensory Feedback" OR "Neurofeedback" OR "Cognitive Remediation" OR "Cognitive Therapy" OR "Relaxation Therapy" OR (MM "Dance Therapy") OR (MM "Play Therapy") OR (MM "Diet Therapy+") OR (MM "Physical Therapy Practice, Research-Based") OR (MM "Physical Therapy+")
12. S10 OR S11
13. S3 AND S6 AND S9 AND S12

CENTRAL

1. MeSH descriptor: [Pregnancy] explode all trees
2. Pregnan* OR Pregnancies* OR Gestation OR Pregnant Wome* OR Pregnant Woman OR Woman, Pregnant* OR Women, Pregnan*
3. birth* OR prenatal OR perinata* OR parturition* OR delivery, obstetri* OR cesarean section* OR extraction, obstetrica* OR labor, induced* OR antenata* OR puerperiu* OR postnata*
4. #1 OR #2 OR #3
5. MeSH descriptor: [Diabetes, Gestational] explode all trees
6. Gestational diabetes mellitus* OR GDM OR gestational diabetes OR pregnancy-induced diabetes mellitus* OR gestational OR pregnancy glucose intolerance OR pregnancy hyperglycaemia* OR pregnancy glycaemic inde* OR Pregnancy-Induced Diabetes OR Diabetes Gestational* OR diabetes in pregnancy OR pregnancy diabetes mellitus* OR gestational, blood glucose metabolism disorder* OR pregnancy in diabetes* OR high risk pregnancy
7. #5 OR #6
8. MeSH descriptor: [Community-Based Participatory Research] explode all trees
9. Community Based Participatory Research* OR Community Based Participatory Research study* OR Community Trial* OR Lay health worker OR community based* OR Health workers OR Health worker OR aides* OR Community based treatment OR community-directed* OR community engagement OR public health program*
10. #8 OR #9
11. non-pharmacological interventions OR Manual Therapy* OR Diet Therapy OR Psychotherapy OR Cognitive Therapy* OR Rehabilitation OR Self Care* OR Physical Therapy OR Physiotherapy OR occupation* OR acupuncture OR social work OR counseling OR nutrition* OR diet OR food OR non-drug OR light therapy OR Snoezelen* OR multimodality therapy* or physical activity or glycemic control monitoring or self-monitoring of blood glucose or health education programme*
12. rehabilitat* OR physiotherapy* or physical therapy* OR cognitive therapy* or behavior therapy* or counseling* or nutrition* or diet* OR Exercise OR exercises OR walking* OR walk OR Stair Climbing OR outdoor* OR Sensory Art Therapies* OR Recreation Therapy OR Behavioral therapy OR Aversive Therapy OR Cognitive Remediation* OR Cognitive Therapy OR Acceptance, Commitment Therapy* OR Relaxation Therapy OR Dance Therapy* OR Play Therapy*
13. #11 OR #12
14. #4 AND #7 AND #10 AND #13
